# Phytochelatins as a Dynamic System for Cd(II) Buffering from the Micro- to Femtomolar Range

**DOI:** 10.1021/acs.inorgchem.0c03639

**Published:** 2021-03-19

**Authors:** Joanna Wątły, Marek Łuczkowski, Michał Padjasek, Artur Krężel

**Affiliations:** Department of Chemical Biology, Faculty of Biotechnology, University of Wrocław, Joliot-Curie 14a, 50-383 Wrocław, Poland

## Abstract

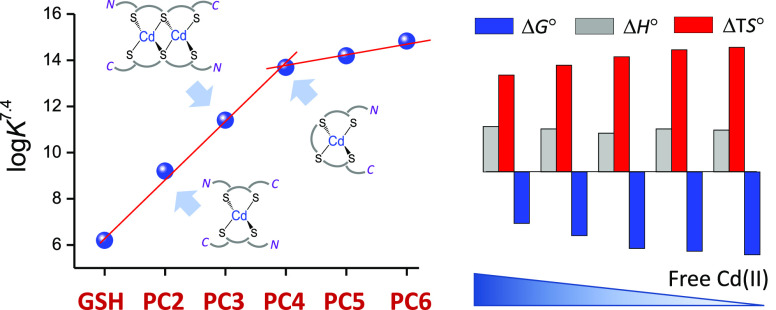

Phytochelatins (PCs) are short Cys-rich peptides with repeating γ-Glu-Cys motifs found in plants, algae, certain fungi, and worms. Their biosynthesis has been found to be induced by heavy metals—both biogenic and toxic. Among all metal inducers, Cd(II) has been the most explored from a biological and chemical point of view. Although Cd(II)-induced PC biosynthesis has been widely examined, still little is known about the structure of Cd(II) complexes and their thermodynamic stability. Here, we systematically investigated glutathione (GSH) and PC2–PC6 systems, with regard to their complex stoichiometries and spectroscopic and thermodynamic properties. We paid special attention to the determination of stability constants using several complementary techniques. All peptides form CdL complexes, but CdL_2_ was found for GSH, PC2, and partially for PC3. Moreover, binuclear species Cd_*x*_L_*y*_ were identified for the series PC3–PC6 in an excess of Cd(II). Potentiometric and competition spectroscopic studies showed that the affinity of Cd(II) complexes increases from GSH to PC4 almost linearly from micromolar (log *K*^7.4^_GSH_ = 5.93) to the femtomolar range (log *K*^7.4^_PC4_ = 13.39) and additional chain elongation does not increase the stability significantly. Data show that PCs form an efficient system which buffers free Cd(II) ions in the pico- to femtomolar range under cellular conditions, avoiding significant interference with Zn(II) complexes. Our study confirms that the favorable entropy change is the factor governing the elevation of phytochelatins’ stability and illuminates the importance of the chelate effect in shifting the free Gibbs energy.

## Abstract 2

Phytochelatins (PCs) are short peptides with repeating γ-Glu-Cys motifs induced by biogenic and toxic metal ions in plants, algae, fungi, and worms. Here, we investigated Cd(II) complexes' formation and determined their stability constants with various methods. Results show that PCs form an efficient system that buffers free Cd(II) ions in the pico- to femtomolar range under cellular conditions. We showed that favorable entropy change is the factor governing the elevation of Cd(II) complexes.

## Introduction

Polycysteine peptides and short proteins play a fundamental role in the metabolism and detoxification of essential and toxic heavy-metal ions.^1,2^ In addition to metallothioneins (MTs), which are small cluster-forming proteins encoded in many genomes from bacteria to humans, phytochelatins (PCs) play similar functions.^2−5^ They are produced by plants, algae, and certain fungi or worms to handle and detoxify heavy-metal ions.^2,6−11^ The major difference from MTs is their polydisperse character. Phytochelatins are noncoded peptides synthesized from glutathione tripeptide γ-Glu-Cys-Gly (GSH) in an enzymatic reaction catalyzed by PC synthase.^3,12,13^ Their primary structure presented as (γ-Glu-Cys)_*n*_-Gly contains *n* repetitions (segments) of the γ-Glu-Cys motif, where *n* varies from 2 to 11 but is generally in the range of 2–5 or 6. The biosynthesis of PCs is initiated by administering a wide range of heavy-metal ions and several anionic species.^2,7^ For example, Cd(II), Pb(II), Zn(II), Sb(III), Ag(I), Ni(II), Hg(II), Cu(II), Sn(II), Au(I), Bi(III), AsO_4_^3–^, and SeO_3_^2–^ induce formation in *Rauvolfia serpentina* cell suspension cultures.^14^ Several studies also show the participation of PGEs (platinum-group elements, such as Pt(II), Rh(III), and Pd(II)) in the synthesis of phytochelatins in some plant organs.^15,16^ Interestingly, inorganic ions induce PC synthesis to different extents; however, Cd(II) demonstrated in many examples induces PC synthesis very efficiently. One more interesting fact about PCs is that their relative amounts depend on metal ions, their doses, and the time of the exposure. The first is synthesis of PC2 from two GSH molecules, then PC3 by incorporating another γ-Glu-Cys building block, and so on. The longer the incubation, the longer the PCs that are produced. However, their production is limited by the concentration of GSH and its biosynthesis, which can significantly decrease under limited sulfur metabolism.^2,3,10,17^ PCs are expected to act as ligands creating reversible complexes for activation of PC synthase and ligands forming stable complexes responsible for the deactivation of heavy metals and the termination of their synthesis.^11^

Even though PCs occur widely in the plant kingdom, relatively little information about the stoichiometries and stabilities of the complexes formed between various PCs has been obtained to date. Despite many analytical problems related to the low air stability of metal–peptide complexes and PCs themselves, many chromatographic (SEC, HPLC) and electrophoretic (CZE) tandem approaches (with electrospray ionization-mass spectrometry (ESI-MS) and inductively coupled plasma mass spectrometry (ICP-MS) have been used to characterize GSH and PC complexes by different techniques.^18−24^ ESI-MS was also used for the characterization of Cd-GSH and Cd-PC complexes from standard mixtures injected directly into the ESI source.^20,25,26^ It is important to note that similar Cd(II) complexes were found despite different buffers being applied. However, the proposed complex stoichiometry was limited to low-molecular-weight (LMW) complexes such as CdL and CdL_2_ for GSH and PC2, and CdL, Cd_2_L, and Cd_3_L for PC2–PC4. Other approaches used were also limited to LMW PCs, such as potentiometry, UV–vis,^27−29^^1^H NMR,^27,30^ EXAFS spectroscopy,^31,32^ ITC,^29,33,34^ and differential pulse voltammetry/polarography.^26,34,35^ Interestingly, spectropolarimetry has never been applied, even though this technique has been successfully used to characterize Cd(II) complexes with MTs.^36,37^ Although minor or significant differences were obtained between each other, all of the applied techniques indicate that mononuclear and polynuclear Cd(II) complexes are formed in the PC system. It cannot be ignored that oligomer formation was postulated for longer PC complexes due to their oxidation.^18,19,23^

Stability studies, in addition to general observations based on spectroscopic investigations, have been limited to just a few reports. In two of them, PC2 was studied potentiometrically, and the ligand was found to bind Cd(II) with a high, sub-nanomolar affinity (log *K*^7.4^ = 9.8 and 10.1).^27,28^ Isothermal titration calorimetry (ITC) studies on various PCs suggested that all of them form Cd(II) complexes with micromolar or sub-micromolar affinity; however, some tendency in stability has been observed. According to these studies, PC4 and PC5 demonstrate the highest Cd(II) affinity from the PC2–PC5 series; however, its sub-micromolar affinity does not match the potentiometric observation about the nanomolar affinity of PC2, which has been identified to be the weakest in an ITC study.^34^ In later spectrophotometric studies, Cd-PC4 was the most stable complex, with log *K*^7.4^ = 7.5, while the constants for PC2, PC3, and PC6 were 6.2, 7.2, and 5.5, respectively.^29^ The same article reports the formation constants determined by ITC, which are slightly lower than those obtained spectrophotometrically.^29^ The molecular reasons for the significant difference between various reports have not been provided, but they may be due to simplifying the system and operating outside the confidence range.^38,39^

**Scheme 1:**
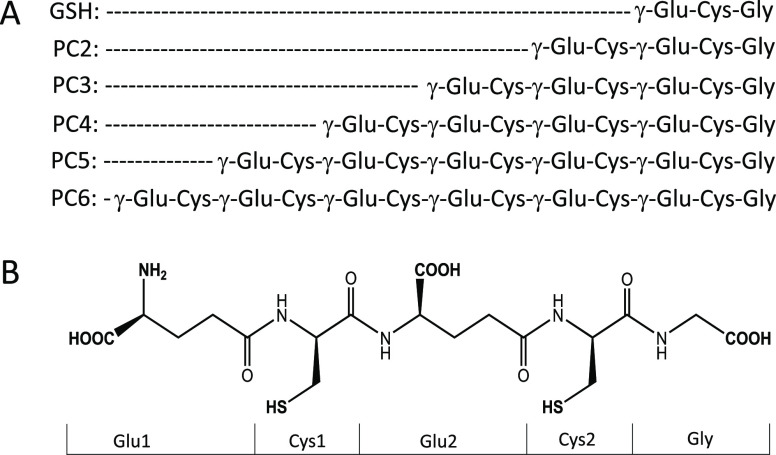
Sequences of γ-Glu-Cys-Containing Peptides Investigated in This Study: (A) Primary Structure of GSH and PCs; (B) Exemplary Structure of PC2 Where Boldface Groups Demonstrate Acid–Base Properties

Here, taking into account the fragmentary knowledge about Cd(II)-to-PCs and GSH interactions, we aimed to systematically investigate Cd(II) complexes of the GSH-PC6 series (Scheme 1) complex formation with the set of spectroscopic (UV–vis, CD) methods, potentiometry, and ITC. However, our main goal is the determination of stability constants with various techniques and deep analysis of the complexation thermodynamics to shed new light on (i) molecular bases of complex formation; (ii) reasons why stability constants differ by several orders of magnitude between various reports; (iii) what drives the increase in stability of longer PCs demonstrated here. Finally, we would like, for the first time, to look at Cd(II)-PC system as an efficient cellular buffer that keeps free Cd(II) concentrations in the low femto- or even subfemtomolar range. The consequence of that is briefly discussed.

## Experimental Section

### Materials

The following reagents were purchased from Sigma-Aldrich (Merck): reduced l-glutathione (GSH, BioXtra, ≥98.0%), (CdSO_4_)_3_·8H_2_O, Cd(CH_3_COO)_2_·2H_2_O, 1,2-ethanedithiol (EDT), thioanisole, anisole, triisopropylsilane (TIPS), nitrilodiaceticpropionic acid (NDAP), triphosphate sodium (TPP), *N*-(2-hydroxyethyl)ethylenediamine-*N*,*N*′,*N*′-triacetic acid (HEDTA), *N*,*N*′-ethylenebis(aspartic acid)trisodium salt (EDDS), and ethylenebis(oxyethylenenitrilo)tetraacetic acid (EGTA). Sodium perchlorate was purchased from Acros Organics. The metal-chelating resin Chelex 100 was acquired from Bio-Rad. Tris(hydroxymethyl)aminomethane (Tris base) and 4-(2-hydroxyethyl)-1-piperazineethanesulfonic acid (HEPES) were obtained from ROTH and BioShop, respectively. *N*,*N*-Dimethylformamide (DMF) and acetonitrile (ACN) were purchased from VWR Chemicals. NaCl, NH_4_HCO_3_, acetic anhydride, diethyl ether, dichloromethane (DCM), and dimethyl sulfoxide (DMSO) were purchased from Avantor Performance Materials Poland (Gliwice, Poland). Tris(2-carboxyethyl)phosphine hydrochloride (TCEP), 1-methyl-2-pyrrolidinone (NMP), *N*,*N*,*N*′,*N*′-tetramethyl-*O*-(1*H*-benzotriazol-1-yl)uronium hexafluorophosphate (HBTU), trifluoroacetic acid (TFA), *N*,*N*-diisopropylethylamine (DIEA), piperidine, TentaGel S Ram, and Fmoc-protected amino acids were obtained from Iris Biotech GmbH (Marktredwitz, Germany). The concentration of metal ion salt stock solutions was 0.05 M and was confirmed by a representative series of ICP-MS measurements. All pH buffers were treated with Chelex 100 resin to eliminate trace metal ion contamination.

### Peptide Synthesis

Phytochelatins were synthesized via solid-phase synthesis on Fmoc-Gly preloaded Wang resin (0.68 mmol/g substitution) using the Fmoc strategy either by manual means or with use of Activo P-11 peptide synthesizer (Activotec). Glutamic acid with a γ-peptide bond was introduced using commercially available Fmoc-Glu(OH)-OtBu (Merck), which allows exclusive formation of a peptide bond with a γ-carboxylate group from the C-terminus. Cleavage and purification were performed as previously described using a TFA/anisole/thioanisole/EDT/TIPS mixture (88/2/2/5/3, v/v/v/v/v) over a period of 2 h followed by 20% CH_3_COOH/CHCl_3_ extraction (PC2, PC3) or precipitation in cold (−70 °C) diethyl ether (PC4–PC6), respectively.^40,41^ The crude peptide was collected by filtration or centrifugation, redissolved in water, lyophilized, and purified using an HPLC system (Waters 2487 or Varian Prostar) on a Phenomenex C18 or Varian Pursuit XRs C 18 column using a gradient of ACN in 0.1% TFA/water from 0% to 40% over 20 min (Phenomenex column) and 0% to 100% over 45 min (Varian column). Purified peptides were identified by an API 2000 ESI-MS spectrometer (Applied Biosystems). The identified peptides and calculated average masses are given in Table S1.

### UV–vis Spectroscopy

UV–vis spectra were recorded using a Jasco V-650 spectrophotometer (JASCO) at 25 °C in a 1 cm quartz cuvette over the range of 200–300 nm.^27,42^ Two spectra were accumulated and averaged. Spectroscopic titrations of 20 μM (PC2), 10 μM (PC3–PC6), and 100 μM (GSH) peptides were performed in chelexed 20 mM (PC3-PC6) or 50 mM (GSH, PC2) Tris·HCl buffer (100 mM NaClO_4_, pH 7.4) with 2.5 mM CdSO_4_ to a final Cd(II) to peptide molar ratio of 4.0. The TCEP was added to a 4–5 molar excess over each cysteine residue as a very weakly metal binding disulfide reducing agent with log *K*^7.4^_ML_ = 2.5, and all titrations were performed under argon atmosphere.^43^ All samples were equilibrated for 2 min after the addition of each portion of Cd(II) stock solution. To confirm that TCEP was sufficient to protect the thiol/thiolate from oxidation during the Cd(II) titration, an additional HPLC examination of the peptides exposed to air for 1 h in the presence and absence of TCEP was performed. The pH titration of GSH and PCs was performed at two metal to peptide molar ratios, 1:1 and 1:2. The pH-dependent formations of the Cd(II) complexes were performed using measurements in the UV range. For that purpose 10 or 20 μM PC peptide solutions containing different amounts of Cd(II) (depending on the M:L molar ratio) were prepared in 0.1 M NaClO_4_, acidified to pH ∼2, and quickly titrated with 0.1 M NaOH in a pH range from 2 to 10 under an argon atmosphere. In the case of GSH a 100 μM solution was used under analogous conditions.

### Circular Dichroism (CD) Spectroscopy

Circular dichroism (CD) spectra of GSH and PCs were recorded using a J-1500 Jasco spectropolarimeter (JASCO) at 25 °C in a 2 mm quartz cuvette, under a constant nitrogen flow over the range of 198–300 nm with a 100 nm/min speed scan. Final spectra were averaged from three independent scans.^44^ Spectroscopic titrations of 10–100 μM peptides with CdSO_4_ were performed in Chelex 20 mM Tris·HCl buffer (0.1 M NaClO_4_, pH 7.4) with the addition of 10 mM TCEP (neutralized to pH 7.4) to 4.0 excess over cysteine residue.^43^ All samples were equilibrated over 2 min under anargon atmosphere after the addition of each portion of 2 mM CdSO_4_ solution. CD signals in mdeg units were converted and analyzed as molar ellipticities (Θ).

### Mass Spectrometry

The binding of Cd(II) to peptides PC2–PC6 and their stoichiometry were monitored in a series of samples of various metal to peptide ratios by ESI-M*S* experiments that were carried out on an amaZon SL ion trap (IT) mass spectrometer (Bruker Daltonik GmbH, Bremen, Germany) in both positive-ion and negative-ion modes. Peptides were dissolved in 10 mM NH_4_HCO_3_ (pH ∼8) to a final concentration varying from 25 μM in the case of PC6 to 50 μM for PC2–PC5. Spectra were measured for metal-free peptides and their mixture with Cd(CH_3_COO)_2_ at metal to peptide ratios of 0.5:1, 1:1, and 2:1.^39,45^ Source parameters were as follows: sample flow, 3 μL/min; ion source temperature, 200 °C; nitrogen flow, 5 L/min at a pressure of 8 psi. Spectra were scanned in the *m*/*z* 100–2200 range. The system was calibrated in positive-ion mode using a ESI-L tuning mix (Agilent Technologies, Santa Clara, California, USA) before acquisitions. Monoisotopic masses, *m*/*z* values, and fragment ion structures were calculated and interpreted using Compass DataAnalysis 4.0 program (Bruker Daltonik) software.

### Potentiometric Titration

The protonation constants of the GSH, PCs, NDAP, and triphosphoric acid and the stability constants of their Cd(II) complexes in the presence of 4 mM HNO_3_ and 96 mM KNO_3_ (*I* = 0.1 M) were determined at 25 °C under an argon atmosphere using pH-metric titrations over a range of 2.5–10.8 (Molspin automatic titrator, Molspin) using standardized 0.1 M NaOH as a titrant. An accurate concentration of NaOH was determined by the titration of a 4.0 mM standard solution of potassium hydrogen phthalate prepared directly before the measurement. Changes in the pH were monitored using a combined glass–Ag/AgCl electrode (Biotrode, Methrom) calibrated daily in hydrogen concentrations using 4 mM HNO_3_ (*I* = 0.1 M).^46^ Sample volumes of 1.7–2.0 mL, a ligand concentration of 0.5–1.5 mM, and Cd(II) to ligand ratios of 0.8:1 to 1:1 were used. The data were analyzed using the Hyperquad program.^47^ The ionic product of the water used in the data processing was 13.80, which represents a 0.1 M ionic strength.^46^

### Peptide Competition with Chelating Agents

In order to determine the Cd(II) to peptide affinity, peptides at 25 (PCs) and 100 μM (GSH) concentrations were equilibrated with various chelating agents forming a 1:1 stoichiometry with Cd(II): TPP, NDAP, NTA, HEDTA, EDDS, EGTA, and CDTA.^48,49^ These competitors were selected in such a way as to cover the −log [Cd(II)]_free_ (pCd) range, where complexation of a particular peptide occurs.^42^ Samples were prepared by mixing an appropriate peptide independently to mixtures with the aforementioned concentrations with a series of metal buffers containing 1 mM chelator with 0.05–0.9 mM Cd(II) over a period of 2 h. Metal buffer sets were prepared in 20 mM Tris·HCl with 100 mM NaClO_4_, 200 μM TCEP, and pH 7.4. The equilibrated samples were measured spectrophotometrically in a 1 cm quartz cuvette in the range of 205–300 nm. The obtained spectra were subtracted from spectra recorded for analogous metal buffers without peptide. The amount of Cd(II) transferred from the metal buffer components to a particular peptide was considered during recalculation of final free Cd(II) values. All -log[Cd(II)]_free_ calculations were performed based on previously or currently established dissociation constants of Cd(II) complexes with chelators using HySS software.^47,50^ All experimental points recorded for each PCs and GSH were fitted to Hill’s equation.^51^

### Isothermal Titration Calorimetry (ITC)

The binding of Cd(II) to GSH and PC peptides was monitored using a NanoITC calorimeter (TA Instruments, USA) at 25 °C with a cell volume of 1 mL. All experiments were performed in HEPES buffer (*I* = 0.1 M from NaCl) at pH 7.4 with 3 mM TCEP used as a non-metal-binding reducing agent.^42,43^ The GSH and PC peptide (titrate) concentrations were 250 and 50 μM, respectively, whereas the Cd(II) (titrant) concentrations were 3 and 0.5 mM, respectively. After temperature equilibration, successive injections of the titrant were made into the reaction cell with 5.22 μL increments at 300 s intervals with stirring at 250 rpm. Control experiments to determine the heats of titrant dilution were performed using identical injections in the absence of titrate. The net reaction heat was obtained by subtracting the heat of dilution from the corresponding total heat of reaction. The titration data were analyzed using NanoAnalyze (version 3.3.0), NITPIC (version 1.2.7),^52,53^ and SEDPHAT (version 15.2b).^54^ First, data were preprocessed using NanoAnalyze software for the Nano-ITC calorimeter. Second, data integration and baseline subtraction were conducted using NITPIC freeware. Afterward, integrated data were fitted with SEDPHAT.

## Results

### Cd(II) Binding to GSH and PC Peptides: Spectroscopic Studies at Constant pH

**Figure 1:**
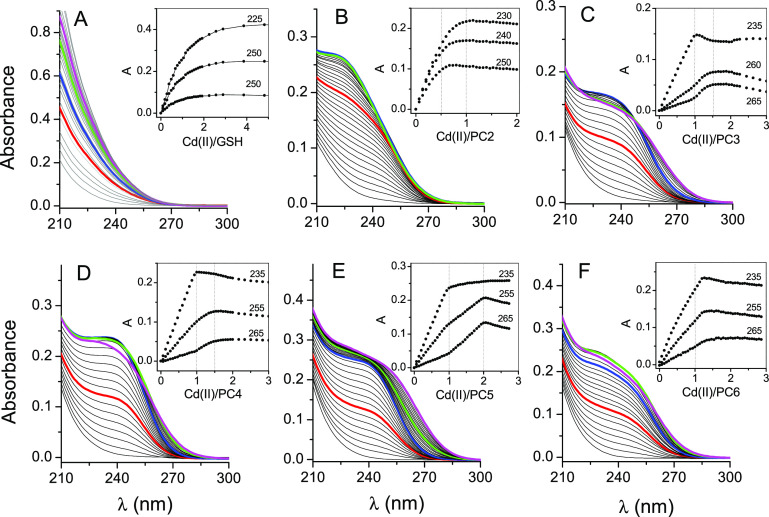
Spectroscopic titrations of GSH (100 μM), PC2 (20 μM), and PC3–PC6 (10 μM) peptides with Cd(II) in 20 mM Tris buffer at pH 7.4, 25 °C (*I* = 0.1 M from NaClO_4_). The insets demonstrate absorbances at particular wavelengths as a function of the Cd(II) to peptide molar ratio. Red, blue, green, and magenta denote 0.5, 1.0, 1.5, and 2.0 molar ratios, respectively.

Due to several, mostly fragmentary reports on Cd(II) binding to short phytochelatins and GSH and a limited number of physicochemical reports on longer PC peptides’ coordination properties, in the first stage of this study, we performed a spectroscopic investigation on the formation of multiple complexes. For that purpose, electronic spectroscopy titration in the UV range was performed at pH 7.4 for all investigated peptides starting from GSH and PC2–PC6 peptides. Figure 1 demonstrates the relations between Cd(II) to peptide molar ratios and absorbance changes at selected wavelengths in the 225–265 nm UV range due to the appearance of characteristic bands corresponding to the formation of ligand to metal charge transfer events (LMCT). The formation of these bands (Figure S1) is typical for the coordination of Cd(II) to sulfur donors. At the same time, their red shift corresponds to the number of sulfur donors in the coordination sphere and the formation of clustered cores where sulfur donors bridge to two independent Cd(II) ions. These spectroscopic tendencies have been described elsewhere for organic compounds, peptides, and Cys-rich proteins.^37,55−57^ UV titration of GSH with Cd(II) reveals the smallest increase of absorbance per mole of added Cd(II), and the isotherm course indicates either a low metal to peptide affinity under the conditions used or the unlikely formation of several complexes of various stoichiometries (Figure 1A). With PC2 as the starting point, absorption inflection points are more pronounced, and in this case, the formation of complexes with CdPC2 and Cd(PC2)_2_ stoichiometry was observed (Figure 1B). No visible red shift of bands was observed for PC2 at higher Cd(II) to peptide ratios, which suggests a lack of significant contribution of multinuclear complex formation. Absorption increase plots for PC3 are significantly different, showing the formation of CdPC3 as the most stable species at low Cd(II) concentration. Addition of Cd(II) continues with absorbance changes that stop at a Cd(II) to peptide ratio of ∼1.5 (Figure 1C). This event accompanies band red shifts, and the observed inflection point suggests the formation of Cd_3_L_2_ species with excess metal. PC4 peptide with four Cys residues predominantly forms the CdPC4 complex with, as is very likely, four sulfur donors bound to Cd(II) in the tetrahedral geometry. In addition to that, the formation of multinuclear species is also possible due to the weaker red shift in comparison to PC3 (Figure 1D). A spectroscopic titration of PC5 reveals the formation of two complexes with stoichiometries CdPC5 and Cd_2_PC5 with an easily visible red shift being characteristic for the second species (Figure 1E). The most obscure situation is observed for PC6. Figure 1F shows one distinct inflection point close to a Cd(II) to peptide ratio of 1:1, indicating the CdPC6 complex. No significant red shift of the LMCT bands was observed for this peptide, in contrast to PC5. The absorption course is here more similar to that of PC4, indicating some similarities between species.

**Figure 2:**
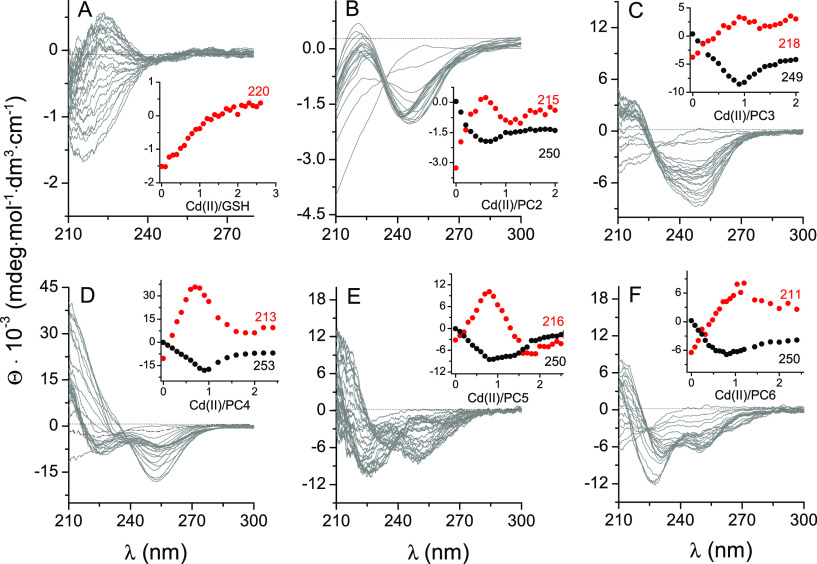
CD spectra of GSH (100 μM) and PC2–PC6 (20 μM) titrations with Cd(II). Spectra were recorded in 20 mM Tris-HCl buffer at pH 7.4 (*I* = 0.1 M from NaClO_4_). The insets present molar ellipticity changes at the indicated wavelengths (values in red and black).

**Figure 3:**
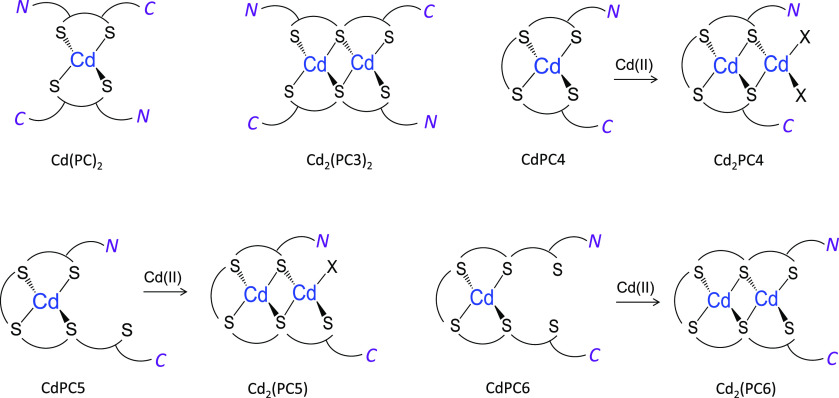
Schematic representation of the most important Cd(II) complexes formed by the series PC2–PC6 with the indication of complex stoichiometry. *N* and *C* denote the N-terminus (γ-Glu residue) and C-terminus (Gly residue) of each PC, respectively. Note that C- and N-termini may be protonated or deprotonated. X represents either donors from the N- or C-terminus or water molecules that fill the coordination sphere of Cd(II).

Circular dichroism spectra of the GSH and PC systems show a picture similar to that of the spectrophotometric results; however, this technique, because of its higher resolution coming from positive and negative Cotton signals, sheds more light on the complexation mode of the examined peptides. It is worth mentioning that phytochelatins and their complexes do not have a well-defined secondary structure and that recorded CD spectra reflect almost solely LMCT signals that evolve upon Cd(II) binding to thiolate donors. Since GSH and PC peptides barely absorb UV light, the CD spectra of metal-free peptides were presented in a raw form (Figure 2). PC2 similarly to GSH demonstrates the formation of the Cd(PC2)_2_ complex, which is observable by the appearance of negative and positive bands at 250 and 215 nm, respectively (Figure 2B). The band at 250 nm is blue-shifted when the Cd(II) to PC2 ratio exceeds 0.5 and stops changing at a ratio of 1.0, which confirms the CdPC2 complex. It has been shown in our previous results, confirmed by NMR measurements, that the Cd(PC2)_2_ complex reveals tetrathiolate coordination from two Cys residues coming from two different PC2 molecules (Figure 3);^27^ however, the additional species CdPC2 is also present, in which Cd(II) is bound by two sulfur donors and nitrogen and oxygen donors coming from the coordination of the N-terminal amine of Glu1 and its α-carboxylate group (Figure S2).^27^ PC3 exclusively forms a complex with a 1.0 Cd(II) to peptide ratio, as reflected by a sharp inflection point (Figure 2C). The shoulder at 232 nm that accompanies the central negative band at 249 nm can be explained by thiolate coordination from three Cys residues and an additional species from other donors constituting a part of the PC3 molecule (Figure S2). It has been shown for PC2 that the carbonyl oxygen of Cys4 or carboxylate of Gly5 may participate in Cd(II) coordination, and those donors are more likely to fill the coordination sphere in CdPC3 species. It is also more likely that at molar ratio 1.0, in addition to CdPC3 species, the Cd_2_(PC3)_2_ complex is formed (Figure 3), which at an excess Cd(II) to peptide ratio turns to Cd_3_(PC3)_2_, which is visible in both absorption and CD spectra (Figures 1 and 2 and Figure S2). Longer PCs, PC4–PC6, fulfill the Cd(II) preferences and provide four sulfur donors, allowing metal ion sequestration with equimolar CdS_4_ species. For that reason, bis complexes were not observed under the conditions used. Their titration with Cd(II) yields the initial formation of equimolar CdL species, followed by transitional Cd_3_L_2_ species formation that finally leads to Cd_2_L (or Cd_4_L_2_) species formation (Figure 3). A comparative analysis of CD spectra acquired for Cd(II) complexes with PC4–PC6 reflects a proportional increase of affinity (see below) toward the binuclear complex formation, where the propensity of the evolution of the negative band at 225–230 nm increases with the number of γ-Glu-Cys repeats (Figure 2D–F). Interestingly, PC5 and especially PC6 do not saturate at a Cd(II) to peptide molar ratio of 2.0, giving room for the formation of binuclear or even trinuclear species in the solution. Spectroscopic studies show that above PC4 phytochelatin complexes become more flexible than PC4, as demonstrated by the possible formation of various polynuclear complexes or even polynuclear oligomers, which has been postulated by others on the basis of chromatographic separation on natural PC sources.^17,21^

### Complexation Monitored by ESI-MS

In addition to identifying peptides and their purity, mass spectrometry can be qualitatively used to monitor the complexation of Cd(II).^45,58^ It is worth noting that this investigation is far from being a quantitative analysis, since the detection of complex ions formed in solution occurs in the gas phase, which changes the relative complex ratio due to their various ionization efficiencies.^59,60^ However, ESI-MS is an suitable method to examine the binding properties and coordination preferences of Cys-rich peptides toward various metal ions, though it has its obvious limitations.^61−65^ Here, MS spectra were collected using samples in NH_4_HCO_3_, which corresponds to the ionization at pH ∼8. Signals and isotopic patterns in the experimental and simulated spectra are perfectly compatible and confirm the correct interpretation. For PC2 (*m*/*z* 540.3, *z* = 1), independently from peptide saturation with Cd(II), only an equimolar CdPC2 species was observable (*m*/*z* 652.1) under the studied conditions (Figure S3). PC3 (*m*/*z* 772.3, *z* = 1; *m*/*z* 386.7, *z* = 2) keeps the same trend at equimolar and subequimolar Cd(II) to peptide ratios (*m*/*z* 884.2, *z* = 1), and is able to yield minor binuclear Cd_2_PC3 species when it is overloaded with metal (*m*/*z* 442.6, *z* = 2) (Figure S4). PC4 (*m*/*z* 1004.3; *z* = 1; *m*/*z* 502.7; *z* = 2) forms a mixture of mono (*m*/*z* 558.7, *z* = 2)- and binuclear (*m*/*z* 613.6, *z* = 2) species at ean quimolar ratio (Figure S5). An even stronger tendency for binuclear species formation is observed for PC5 (*m*/*z* 618.8, *z* = 2), where at an equimolar ratio, these complexes (*m*/*z* 729.6, *z* = 2) were detected, while with excess metal minor trinuclear species (*m*/*z* 784.6, *z* = 2) in addition to the major binuclear species are formed (Figure S6). PC6 (*m*/*z* 734 8, *z* = 2) preferentially forms binuclear species (*m*/*z* 845.6, *z* = 2) (Figure S7). Trinuclear species were not formed in that system using ESI-MS under the applied conditions.

The ESI-MS results reveal that equimolar CdL complexes are formed for PC2 and PC3, while the longer homologues PC5 and PC6 preferably form binuclear Cd_2_L complexes. PC4, containing four γ-Glu-Cys segments, constitutes the transition point, and while it preferentially forms mononuclear species, it still exhibits some ability to bind two Cd(II) atoms within a single peptide moiety. Nevertheless, mass spectrometry data did not fully confirm the spectroscopic findings, neither the presence of bis complexes (CdL_2_ and Cd_2_L_3_) for PC2 and PC3 nor cluster species (such as Cd_3_L_2_) for longer peptide molecules (PC4–PC6) that may form binuclear species even at an equimolar ratio. Knowing the limitations of mass spectrometry measurements, determined by the fact that the species are detected in the gas phase, we may assume that the lack of bis and tris ligand species in dynamic labile systems such as phytochelatin homologues does not prove their absence in solution.^59,66^

It has been shown in a direct analysis of plant extracts (*Datura innoxia*) using nano-ESI-MS/MS and capillary LC/ESI-MS/MS methods that PC5 phytochelatin forms CdPC5 species in addition to Cd_2_PC5 and Cd_3_PC5 complexes. PC3, similarly to our studies, forms CdPC3 and Cd_2_PC3 species, while only the CdL complex was identified for PC2 and PC4.^24^ These observations made for natural products are highly convergent with our spectroscopic results. In another report on extracts from *Arabidopsis thaliana* exposed to Cd(II), the authors using SEC-ICP-MS and CZE-ICP-MS found that PC2 forms CdPC2, Cd_3_(PC2)_2_, and Cd_4_(PC2)_2_ complexes.^21^ In this and previous studies on PC2 metal binding properties, we could not find multinuclear species for PC2. Still, it cannot be excluded that they can be formed in plants when the Cd(II) concentration increases, and other PCs are not yet available. According to the same authors, PC3 and PC4 form only CdL complexes. With regard to glutathione, the same group and others have presented that, under ESI-MS experimental conditions, not only CdGSH but also Cd(GSH)_2_, Cd(GSH)_3_, and Cd(GSH)_4_ species are formed.^20,67^ Spectroscopic and potentiometric investigation performed in water solution for the relatively well characterized Cd(II)–GSH system showed the formation of only CdGSH and Cd(GSH)_2_ complexes.^68−70^ However, for higher reactant concentrations, Cd(GSH)_3_, and Cd(GSH)_4_ complexes have been observed in ^113^Cd NMR and EXAFS studies.^71^ This nicely demonstrates that Cd(II) complexes with γ-Glu-Cys segments may form a series of equilibria, according to which a relative molar ratio of particular species depends on the reactant ratios and concentrations. Depending on that, species present at low fractions under the applied conditions are beyond the detection limit.

### Acid–Base Properties of GSH and PCs

Knowledge of the acid–base properties of the ligands is essential for the investigation of stability constants, especially over a wide range of pH. Protonation constants of GSH and some PCs and their derivatives have been determined in the past; however, this knowledge is not complete, and the constants were determined under various experimental conditions. All peptides investigated here were characterized potentiometrically at 25 °C and 0.1 M ionic strength to unify the conditions. The obtained protonation log β_*ij*_ and corresponding p*K*_a_ values are presented in Table 1. For PC6, the most acidic protonation constants were not determined due to the limitation of the potentiometric method, which uses a standard pH range from ∼2.5 to ∼11.

**Table 1:** Protonation Constants of GSH and PC Peptides Determined Potentiometrically at 25 °C (*I* = 0.1 M from KNO_3_)^a^

	log β_*jk*_^b^					
species	GSH	PC2	PC3	PC4	PC5	PC6
HL	9.65(1)	10.25(5)	10.21(2)	10.20(3)	10.26(2)	10.45(2)
	*9.65*	*10.25*	*10.21*	*10.20*	*10.26*	*10.45*
H_2_L	18.41(1)	19.94(2)	19.52(2)	20.14(2)	20.39(2)	20.73(2)
	*8.76*	*9.69*	*9.31*	*9.94*	*10.13*	*10.28*
H_3_L	22.05(1)	28.53(2)	28.77(1)	29.49(3)	29.99(3)	30.53(4)
	*3.64*	*8.59*	*9.25*	*9.35*	*9.60*	*9.80*
H_4_L	23.30(2)	32.83(3)	37.08 (1)	38.59(2)	39.32(2)	40.18(2)
	*1.95*	*4.30*	*8.31*	*9.10*	*9.33*	*9.65*
H_5_L		36.00(2)	41.13(2)	46.99(2)	48.30(2)	49.39(2)
		*3.17*	*4.05*	*8.40*	*8.98*	*9.21*
H_6_L		38.43(5)	44.70(2)	51.40(3)	56.68(1)	58.38(1)
		*2.43*	*3.57*	*4.41*	*8.38*	*8.99*
H_7_L			47.48(2)	55.04(2)	61.26(3)	67.21(1)
			*2.78*	*3.64*	*4.58*	*8.83*
H_8_L			48.93 (7)	58.47(3)	65.22(2)	71.98(1)
			*1.45*	*3.43*	*3.96*	*4.77*
H_9_L				61.10(2)	68.75(3)	76.05(2)
				*2.63*	*3.53*	*4.07*
H_10_L				nd	72.09(2)	79.88(2)
					*3.34*	*3.83*
H_11_L					74.70(3)	83.37(3)
					*2.61*	*3.49*
H_12_L					76.46(5)	86.34(4)
					*1.76*	*2.97*
H_13_L						88.99(5)
						*2.65*
H_14_L						nd

a Constants are presented as cumulative log β_*j*__*k*_ values. Standard deviations of the last digits are given in parentheses, at the values obtained directly from the experiment. L stands for a peptide with acid–base active groups. Values in italics correspond to p*K*_a_ values of the peptides and were derived from cumulative constants. nd denotes not detectable under the conditions used. log β(H_*j*_L_*k*_) – log β(H_*j-1*_L_*k*_) = p*K*_a_.

b β(H_*j*_L_*k*_) = [H_*j*_L_*k*_]/([H]^*j*^[L]^*k*^), in which [L] is the concentration of the fully deprotonated peptide.

The glutathione molecule contains four groups with acid–base properties (Scheme 1 and Table 1) for which p*K*_a_ values obtained here correspond very well to previously determined constants under the same conditions.^50,72^ The most acidic is the α-carboxylic group of glutamic acid (p*K*_a1_ = 1.95). Slightly more basic is the carboxylic group of the Gly residue with p*K*_a2_ ≈ 3.64. The C-terminal carboxylate has a significant effect on the acid–base properties in GSH, as it forms a salt bridge with a thiol group and, less preferentially, with a positively charged amine.^72^ This causes a slight increase in the thiol group’s basicity, demonstrated by p*K*_a3_ = 8.76. ^1^H NMR data have shown that thiolate forms a salt bridge with a positively charged amine, increasing its basicity, manifested by p*K*_a4_ = 9.65 (Figure S8).^56,72^

PC2, being an elongated glutathione molecule by the γ-Glu-Cys segment from the N-terminus, contains six acid–base-active groups (Scheme 1): three carboxylic, two thiols, and an α-amine. Their p*K*_a_ values determined here potentiometrically (Table 1) are quite convergent with previously obtained data in 0.1 M KNO_3_^27^ and less convergent for those obtained in 1.0 M KNO_3_^28^ due to the significant difference in ionic strength present during potentiometric titrations. The acidic p*K*_a_s are 2.43, 3.17, and 4.30, and according to previous NMR data, they correspond to αGlu1, αGlu2, and Gly5 carboxylic groups, respectively.^27^ The basic p*K*_a_s are 8.59, 9.69, and 10.25, and they can be assigned to thiols of Cys2, Cys1, and the amine group of Glu1, respectively. However, it is worth noting that these values are macroconstants, and group constants can be determined only by NMR spectroscopy.^27^ Indeed, in comparison to group constants (p*K*_a_^SH^) of Cys1 and Cys2 thiols, they are much closer to each other and are 9.53 and 9.40, respectively. At the same time, the amine function is manifested by p*K*_a_^NH3+^ = 10.01, according to our previous study (Figure S8).^27^ This picture shows that the elongation of the peptide chain from GSH to PC2 causes an increase in the basic groups’ acidity due to a higher number of negatively charged carboxylates present in the molecule. They affect the acidity of thiols through the induction effect and salt bridge formation, similarly to GSH.^56^

Additional step-by-step elongation from PC2 to PC6 causes only a minor increase in thiol and amino function basicity due to the continuous growth of the quantity of thiol and α-Glu carboxylate groups in γ-Glu-Cys segments. Their p*K*_a_ values vary in the range 8.3–10.5 (Table 1 and Figures S8 and S9). Previous NMR studies^30^ on PC derivatives with an acetylated amino group showed that the addition of each γ-Glu-Cys segment increases the average group constant of the thiol by ∼0.11 log unit; however, the transformation from PC2 to PC3 is manifested by an increase of 0.37 of logarithmic value.^30^ This demonstrates that all PC peptides are flexible molecules with numerous intramolecular interactions (e.g., SH/S^–^···NH_3_^+^), as proven for GSH.^72^

Overall, potentiometric data supported by previous NMR investigations show that there is no significant difference in acid–base properties among the GSH and PC2–PC6 series other than a minor increase in the thiol basicity with the length of the PCs. Therefore, the question is how the acidity of thiols changes when Cd(II) competes with protons and ligands coordinated with the metal ion.

### Considerations of the Formation of Cd(II) Complexes and Their Stability

**Figure 4:**
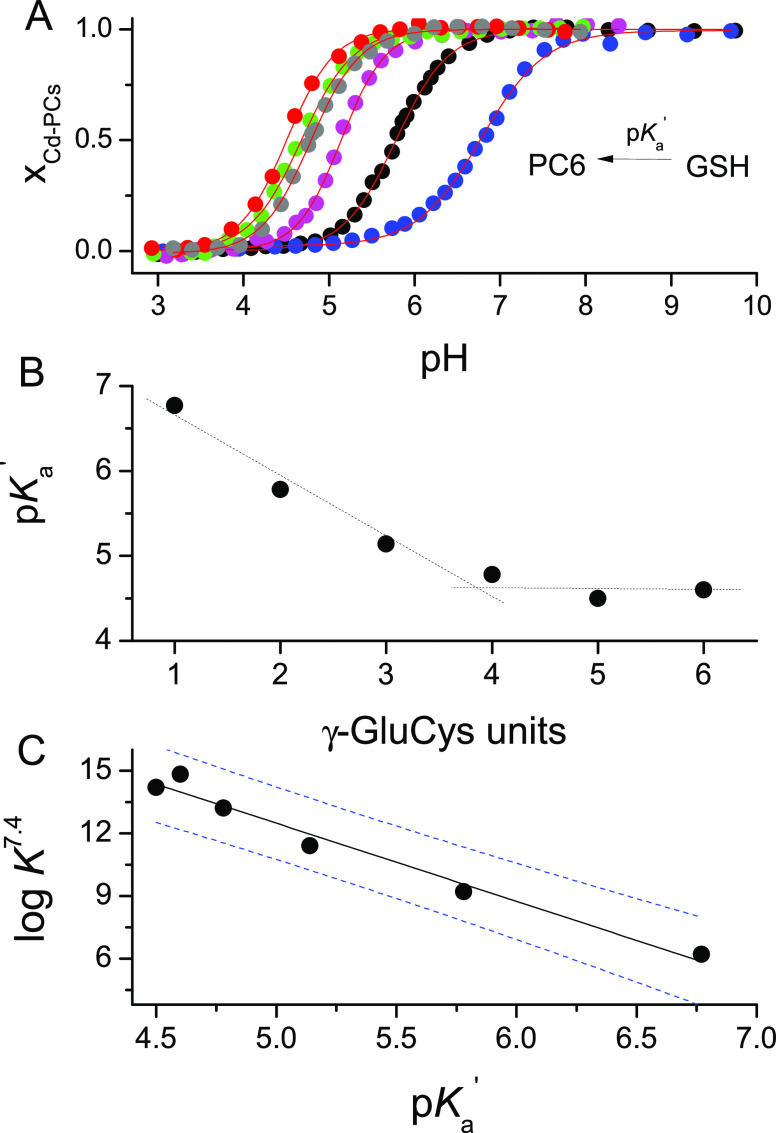
pH-dependent relations of Cd(II) complex formation. (A) Isotherms of Cd-GSH and Cd-PCs system formation as a function of pH (metal to peptide molar ratio 1:1). Molar fractions (*x*_Cd-PCs_) were calculated from the absorbance at a specific wavelength characteristic for the particular PC system. p*K*_a_′ denotes the inflection point, which corresponds to 50% complex formation of a particular ligand. (B) Dependence of p*K*_a_′ values on the number of γ-Glu-Cys segments in the GSH and PC2–PC6 series. (C) Linear relation between the apparent log *K*^7.4^_Cd-L_ constants and p*K*_a_′ values (*R* = 0.99, *R*^2^ = 0.97). Dashed blue lines indicate the 95% confidence interval.

To evaluate the acidity of thiols in the presence of Cd(II) ions, we spectrophotometrically titrated GSH and PC2–PC6 (1:1 molar ratio) over a wide range of pH. Figure 4A demonstrates the isotherms of Cd(II) complex formation. Inflection points correspond to p*K*_a_’ values which are averaged dissociation constants for the thiols under the applied conditions. The p*K*_a_′ values decrease from GSH (6.8) to PC4 (4.6) significantly, while they become almost constant from PC4 to PC6 (Figure 4B). This is a clear indication of changes in the affinities of particular ligands that are conversely related to p*K*_a_ values. An increased acidity of the Cys residue results in its partial dissociation at neutral pH, which decreases the negative effect of Cys deprotonation on complexation and promotes its binding to Cd(II). However, a direct and comprehensive assessment of PC affinities toward Cd(II) without a consideration of ligand protonation is impossible.

Potentiometry is one of the most precise methods for determination of stability constants of small peptides due to its sensitivity and possibility to obtain a stoichiometric model over a wide range of pH. An evaluation of the speciation profile and assigned stability constants over a wide pH range could translate into a more exoteric and widely appreciated apparent affinity constant. Alternatively, as is often the case in more complicated systems, competivity indexes might be calculated.^73^ The Cd(II) binding affinities of GSH and individual phytochelatin peptides, revealed as stability constants, have been evaluated potentiometrically and are presented in Table 2. Although Cd(II) complexes with GSH have been characterized potentiometrically in the past,^69,74^ we reevaluated these investigations to compare the obtained data with those of PCs under the same conditions and with the same instrumentation. Cd(II), similarly to Zn(II), forms with GSH equimolar CdH_2_L, CdHL, CdL, and CdH_–1_L and CdH_3_L_2_, CdH_2_L_2_, CdHL_2_, CdL_2_, and CdH_–1_L_2_ bis complexes with variously protonated GSH molecules (Figure S10).^75^ No higher stoichiometries were obtained by experimental data fitted and observed under the conditions used, which does not exclude the presence of Cd(GSH)_3_ and Cd(GSH)_4_ species in minor amounts as observed in Cd K-edge and L3-edge X-ray absorption spectra recorded for higher reactant concentrations and Cd(II) molar ratios over GSH.^71^ According to the obtained potentiometric model, the most predominant complex at neutral pH is CdHL_2_ (at higher GSH to Cd(II) molar ratios), which turns to CdHL species under equimolar conditions. A similar observation regarding the stoichiometry was made by other groups.^50,70,71,74,76−78^

**Table 2:** Cd(II) Stability Constants of GSH and PC Peptide Complexes Determined Potentiometrically at 25 °C (*I* = 0.1 M from KNO_3_)^a^

	log β_*ijk*_^b^				
species	GSH	PC2	PC3	PC4	PC5
Cd_2_H_5_L					62.33(7)
Cd_2_H_3_L				47.99(3)	53.90(5)
Cd_2_H_2_L			37.36(7)		48.85(4)
Cd_2_HL			33.18(5)	38.87(3)	
Cd_2_L			28.29(7)		
CdH_4_L					
CdH_3_L					46.91(4)
CdH_2_L	21.58(4)	27.50(3)		37.2(3)	40.23(5)
CdHL	16.20(2)	22.82(1)	26.79(4)	30.63(2)	30.69(6)
CdL	9.00(1)	16.14(2)	17.86(5)	20.99(2)	20.68(6)
CdH_–1_L	–0.96(3)		7.38(3)	9.83(4)	
CdH_3_L_2_		48.10(7)			
CdH_2_L_2_	32.04(5)	41.25(5)			
CdHL_2_	24.22(1)	31.72(2)			
CdL_2_	15.05(2)	21.35(4)			
CdH_–1_L_2_	4.50(3)				

a Constants are presented as cumulative log β_*i*__*j*__*k*_ values. L stands for a fully deprotonated peptide ligand that binds Cd(II). Standard deviations of the last digits are given in parentheses, at the values obtained directly from the experiment.

b β(M_*i*_H_*j*_L_*k*_) = [M_*i*_H_*j*_L_*k*_]/([M]^*i*^[H]^*j*^[L]^*k*^), in which [L] is the concentration of the fully deprotonated peptide.

**Figure 5:**
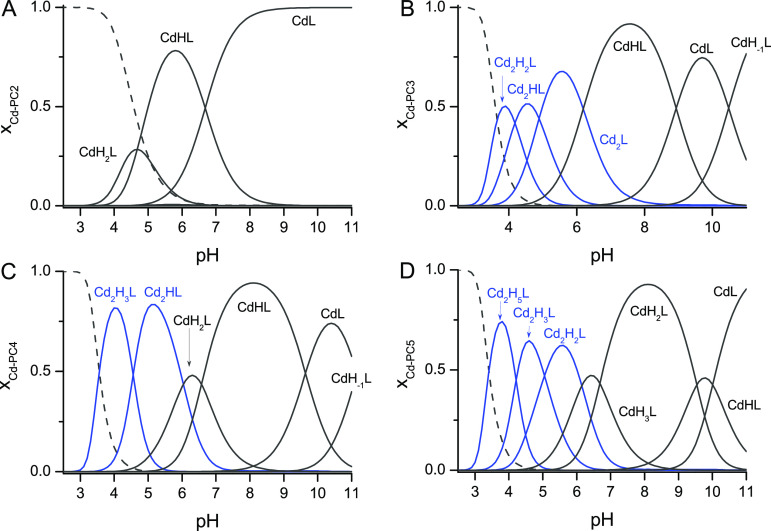
Species distribution profiles for Cd(II) complexes of PC2 (A), PC3 (B), PC4 (C), and PC5 (D) at a 1.0 Cd(II) to peptide ratio 1.0 (500 μM Cd(II) and 500 μM PCs) on the basis of potentiometric results (25 °C, *I* = 0.1 M from KNO_3_). Dashed, blue, and dark gray lines correspond to free Cd(II), binuclear, and mononuclear species, respectively. For clarity GSH and other metal to peptide ratio plots are presented in Figures S10 and S11 in the Supporting Information.

The potentiometric model of the Cd(II)–PC2 system shows some analogy to the Cd(II)–GSH system mostly because of the formation of bis complexes that does not occur for longer PCs. The species CdH_2_L_2_, CdHL_2_, and CdL_2_ that contain Cd(II) as a tetrathiolate in a tetrahedral geometry are present over a wide range of pH (at an of excess PC2 with respect to Cd(II)) and differ in amine group protonation. Under equimolar conditions, CdHL and CdL species dominate at neutral pH (Figure 5A). Our previous ^1^H NMR studies have shown that this species contains the {O_2_S_2_} and {NOS_2_} donor patterns in the Cd(II) coordination sphere, respectively, which makes PC2 similar to GSH but only at a Cd(II) to PC2 ratio of lower than 0.5.^27^ The literature is lacking in X-ray spectroscopic studies on pure PC2 complexes with Cd(II). Available EXAFS data for a mixture of PCs from Cd(II)-treated cell suspension cultures of *Rauvolfia serpentina* show Cd(II) in a tetrathiolate coordination, which corresponds well with our model. It is worth noting that cell samples contained only a few percent of PC2 in the total mixture.^31^ In general, potentiometric data are convergent with spectroscopic results regarding the stoichiometric model. Although PC2 possesses some coordination similarities to GSH, it is important to note that it forms significantly more stable complexes (see below), which, with excess ligand, demonstrate tetrathiolate coordination in contrast to GSH.

Extension of PC2 to PC3 changes the peptide’s coordination properties due to its higher number of donors; however, some similarities in Cd(II) binding are also observed in this case. Potentiometric results (Table 2) indicate that two different kinds of complexes are present in equilibrium at equimolar substrate concentration, and both of them demonstrate a stoichiometry 2:1 and 1:1. The species distribution plot (Figure 5B and Figure S11) shows that the binuclear species Cd_2_H_2_L, Cd_2_HL, and Cd_2_L and mononuclear species CdHL and CdL are formed ove a wide range of applied pH and differ as in the case of PC2 in the protonation of the amine group of Glu1. The CdH_–1_L complex contains a coordinated hydroxyl group, as observed for GSH. This model is convergent with spectroscopic results performed at pH 7.4, where the complex with a stoichiometry of 1:1 (or 2:2) is more likely preferred, and such complexes are formed at an excess of ligand to Cd(II). High Cd(II) preference to form tetrathiolate species is feasible in the case of binuclear complexes where two metal ions are surrounded by four Cys residues, one of which is bridging to two metal ions (Figure 3). What is more similar to PC2 is a CdL species with three sulfur and one oxygen or nitrogen providing a tetrahedral environment around metal ion (Figure S2). Interestingly, the addition of Cd(II) to equimolar species results in a red shift of the LMCT band and an additional absorbance increase up to a Cd(II) to peptide molar ratio of 1.5 (Figure 1C). This inflection point indicates the formation of the Cd_3_L_2_ complex, the presence of which was also postulated on the basis of a CD titration (Figure 2C). Thus, potentiometric results are lacking in these species, as the potentiometric titrations were performed at an equimolar ratio of PC3 to Cd(II).

The PC4 peptide is different from the previously described peptides due to the number of Cys residues. Four cysteines, well separated in peptide chain sulfur donors, can form a CdL complex with a tetrathiolate CdS_4_ center. This formation is clearly observable in UV, CD, and ESI-MS titrations (Figure 1D). Additionally, the potentiometric model shows that the CdHL complex predominates at neutral pH (Figure 5C). The CdH_2_L species at slightly acidic pH contains either one protonated thiolate or protonated carboxylate (e.g., of Gly9), and the CdH_–1_L species present in alkaline conditions is more likely a complex with a coordinated hydroxyl group. In addition to mononuclear complexes, binuclear species are suggested to be formed by the potentiometric model: namely, Cd_2_H_3_L and Cd_2_HL (Figure 5C and Figure S11). Spectroscopic results presented in Figure 1D and Figure 2D show that a clustered species is formed and remains stable above a Cd(II) to PC4 ratio of 2.0. However, on the basis of the number of available donors in PC4, the participation of nitrogen and oxygen donors in Cd(II) coordination is highly possible (Figure 3). Optionally, possible complex oligomerization (see below) may increase the availability of sulfur donors due to their bridging. Interestingly, PC5 and PC6 peptide coordination features are similar to those PC4, although we could not fit potentiometric data for PC6 due to the system complexity and software limitations. In both cases with equimolar or an excess of ligand to Cd(II) mononuclear, variously protonated complexes are formed (Figure 5D). The most predominany species in the case of the Cd(II)–PC5 system is CdH_2_L, with a Cd(Cys)_4_ core (Figure 3), which more likely contains a protonated amine and one thiol group. The dissociation of those groups results in the formation of CdHL and CdL complexes. When additional Cd(II) is added, the formation of binuclear complexes is more likely (Figure S11). This phenomenon is clearly visible in CD spectra (Figure 2E,F), where characteristic isoelliptic points are present for PC5 and PC6. Such a point was much less visible for PC4. According to that, the intensity of the negative signal at 219 nm increases with PC length starting from PC4. The intensity of the negative signal at 250 nm decreases at the same time. The observable difference may be connected to conformational changes in PC chains or separation of LMCT bands due to their physicochemical character. It is possible and even suggested by SEC studies that PCs may form oligomeric species with an excess of Cd(II) with specific fractions of bridging sulfur donors.^21^ To conclude this part, it is essential to underline that the predominant and the most stable species at neutral pH are those with isolated tetrathiolate CdS_4_ centers, other than PC3, where a clustered center is formed due to bridging sulfur donors.

### Evaluation of Potentiometric Stability Data: Apparent Constants and Competivity Indexes

**Figure 6:**
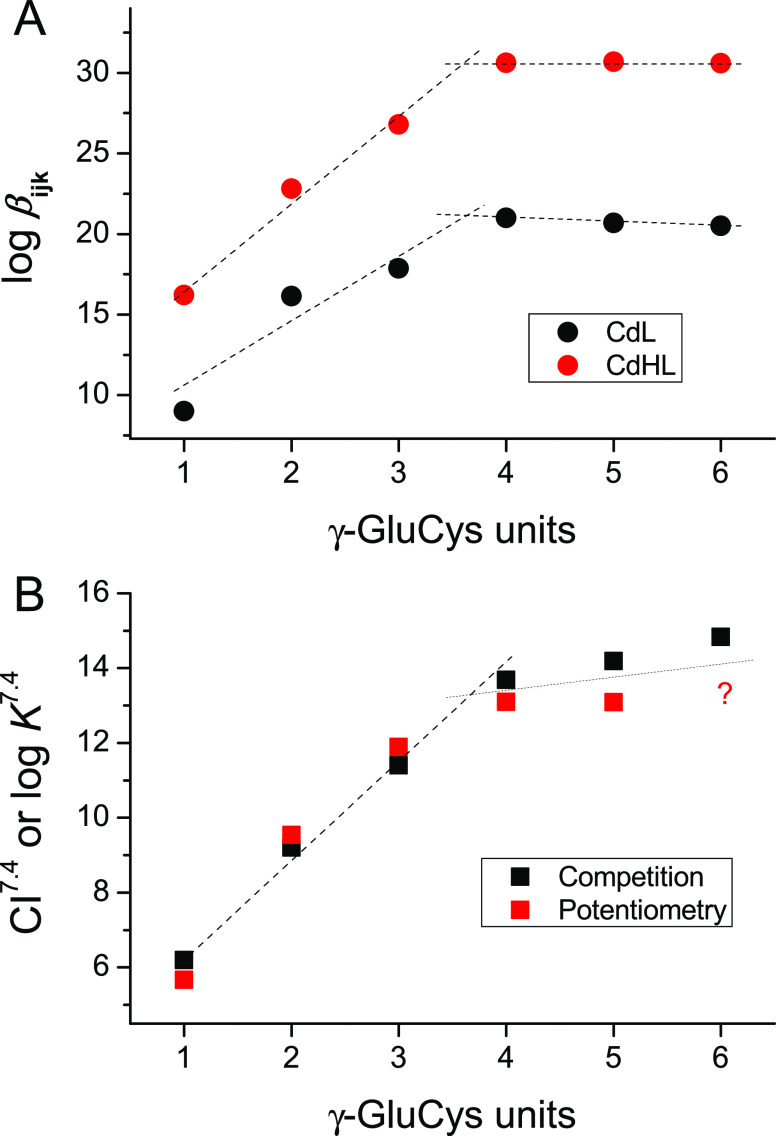
Relation of stability constants of Cd-PC complexes depending on the number of γ-Glu-Cys segment repeats in the peptide. (A) Comparison of cumulative constants of CdHL and CdL complexes derived from potentiometry. Compared complexes were detected for all investigated peptides. (B) Comparison of the formation constant (log *K*^7.4^) determined in the competition experiments with complexones and competitivity indexes (CI^7.4^) derived from the potentiometric data. CI values used here were calculated for concentrations used in potentiometric experiments (Table 3).

A quantitative comparison of PC binding affinity can be made using apparent constants that are valid for specific conditions, and their values are not affected by differences in group acidity among the compared ligands. To calculate the formation constant at the same pH (e.g., pH 7.4), one needs to determine first the concentrations of substrates and products being at equilibrium at this pH. In such calculations, concentrations of differently protonated 1:1 complexes are added together to obtain the total complexed and free ligand concentrations.^41^ Although this procedure is easy, it is not valid for the GSH and PC systems due to complexes with various metal ions and ligands (stoichiometries other than 1:1). Apparent formation constants can be calculated and compared only for the same stoichiometries. To avoid this inconvenience, we calculated here competitivity indexes (CI, here CI^7.4^, valid for pH 7.4), rather than apparent constants, defined by eqs 1 and 2 at constant metal to ligand ratios (L 500 μM and Cd(II) 400 μM as in potentiometric analysis and L 500 μM and Cd(II) 200 μM to promote formation of the most stable complexes with a tetrathiolate coordination), where different stoichiometries are simplified to a 1:1 stoichiometry. Due to this simplification CI values are appropriate to compare various ligands prone to form various stoichiometries with the analyzed metal ion and have been successfully used in the past for the comparison of chemically different ligands and macromolecules.^42,47,73^
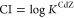
1
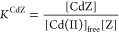
2To calculate the CI value, one needs to define first CdZ, which is a Cd(II) complex of the theoretical molecule Z and CdZ is ∑_*ijk*_Cd_*i*_H_*j*_L_*k*_ at a given overall component concentration. Z is, therefore, ∑_*jk*_H_*j*_L_*k*_ under the applied conditions. Calculated CI^7.4^ values of GSH and PC2-PC5 are given in Table 3, and their comparison as a function of the number of γ-Glu-Cys repeating segments is plotted in Figure 6B. This comparison agrees with the observations made for a coarse analysis based only on the cumulative stability constants of CdHL and CdL complexes across the analyzed peptides (Figure 6A). The minor difference (if any) between CI^7.4^ values obtained for different reactant concentrations comes from different stoichiometries of complexes between various ligands and their various fractions at a particular ratio. Determined CI^7.4^ values show that GSH, PC2, and PC3 form Cd(II) complexes with micromolar, sub-nanomolar, and low picomolar affinities. PC4–PC6 demonstrate similar femtomolar affinities toward Cd(II). The affinity difference between the weakest (GSH) and the strongest Cd(II) complex (PC4, PC5) is more than 7 orders of magnitude in a formation constant, which transforms to a vast ΔΔ*G*° value of less than −10 kcal/mol, and this corresponds to ∼−3.4 kcal/mol of stabilization Gibbs free energy effect per γ-Glu-Cys segment in the GSH and PC2–PC4 series.^42^

**Table 3:** Comparison of Apparent Formation Constants and Competitivity Indexes (CI)^a^ Calculated for Cd(II) Complexes of GSH and PC Peptides on the Basis of Spectroscopic Competition and Potentiometric Titrations, Respectively

	CI^7.4^ (ligand/Cd(II))				
ligand	500 μM/400 μM	500 μM/250 μM	50 μM/50 μM	log *K*^7.4^	av log *K*^7.4^
GSH	5.76	6.14	5.67	6.20	5.93
PC2	9.94	10.19	9.54	9.20	9.37
PC3	11.87	11.87	11.89	11.40	11.64
PC4	13.26	13.26	13.10	13.69	13.39
PC5	13.18	13.18	13.09	14.19	13.64
PC6				14.83	

a CI is the logarithm of the apparent dissociation constant of CdL complex (Cd(II) complex of theoretical molecule Z), such as [CdZ] = ∑_*ijk*_[Cd_*i*_H_*j*_L_*k*_] at the given overall component concentrations. The concentrations of Z were set at 1 mM and those of Cd(II) at 0.25 mM.

### Apparent Constants Determined by the Competition with Chelating Agents

As mentioned in the previous section, stability constants obtained from direct metal to ligand titrations may be seriously underestimated in the case of ligands that bind a metal ion with nanomolar or higher affinity.^48^ It has been shown in several examples that the application of competitive ligands with an affinity slightly lower than or similar to that of a ligand of interest helps in the accuracy of determining stability constants.^48,79^ If spectroscopy is used for metal equilibrium monitoring, applied chelators should not interfere with the analyzed signal. Due to the intense signals of LMCT bands in the UV range occurring upon Cd(II) binding to thiolates of GSH and PCs, we chose their intensity analysis to determine apparent formation constants in order to compare them to those obtained from potentiometry (CI values, Table 3). To do so, the investigated peptides were incubated with Cd(II) and selected chelating compounds that do not absorb significantly in the UV range at pH 7.4. The range of Cd(II) affinity of those compounds varied from micromolar to the low femtomolar range, and they were chosen on the basis of CI^7.4^ values derived from potentiometry (Figure 6B). This list includes CDTA (log *K*^7.4^_CdL_ = 14.92), EGTA (log *K*^7.4^_CdL_ = 13.10), HEDTA (log *K*^7.4^_CdL_ = 10.68), EDDS (log *K*^7.4^_CdL_ = 8.28), and NTA (log *K*^7.4^_CdL_ = 7.53).^80^ To monitor Cd(II) binding to the weakest ligands such as GSH and PC2, we used pentasodium triphosphate (TPP [acid form], log *K*^7.4^_CdL_ = 6.35) acid and NDAP (log *K*^7.4^_CdL_ = 6.08), for which stability constants were determined here potentiometrically due to some inconsistency in the literature (Table S2 and Figure S12). Overall, the set of chelating agents used allowed us to cover a large range from micromolar to sub-femtomolar concentrations of free Cd(II) that were strictly controlled (Figure S13). It is worth noting that, during equilibration, chelators compete with peptides for Cd(II), and the most stable complexes of GSH and PCs are formed primarily due to Cd(II) limitation in buffered media.

**Figure 7:**
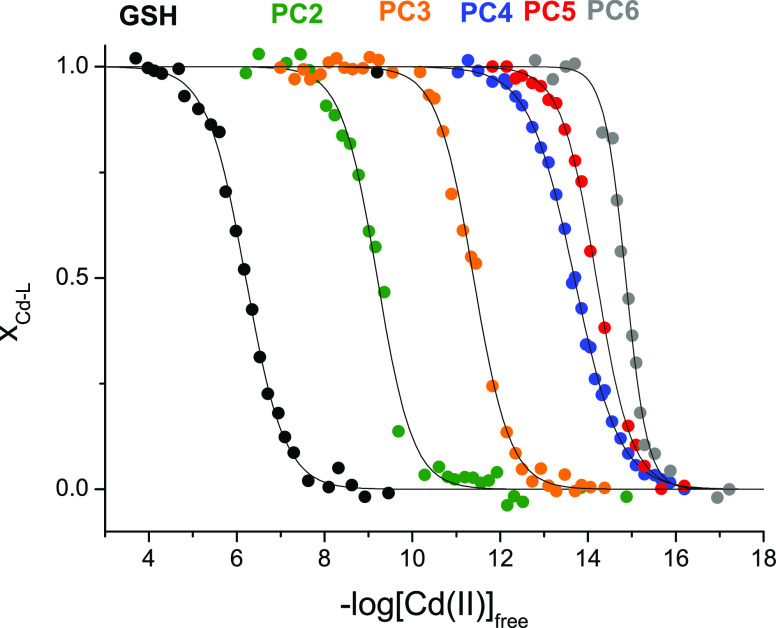
Isotherms of the Cd(II)-L complex formation in the series of GSH and PC2–PC6 as a function of free Cd(II). The free Cd(II)-controlled buffers (50 mM TES, 0.1 M NaClO_4_) contained series of metal chelators of various Cd(II) affinities with the gradual saturation of Cd(II) (see the Experimental Section). Molar fractions (*x*_Cd-PCs_) were calculated from the absorbance at a specific wavelength characteristic for a particular PC system. Inflection points correspond to the conditional log *K*^7.4^_CdL_ value.

In order to determine the apparent formation constants of Cd(II) complexes with GSH and PCs, absorbance intensities were plotted against free Cd(II) concentration (−log [Cd(II)]_free_ = pCd) calculated on the basis of the known affinity of the competitive ligand to Cd(II). Then, intensities were normalized to the 0–1 range and free Cd(II) concentrations were corrected for metal transfer from the chelating ligand to the peptide during equilibration. All data were finally fitted to Hill’s logarithmic equation (Figure 7) and are presented in Table 3.^42,48^ The constants obtained are in the same range as competitivity indexes calculated exactly for the same concentration of peptides used in the spectroscopic competition. Depending on the peptide values, log *K*^7.4^_CdL_ and CI^7.4^ differ not more than ±1 logarithmic unit (Figure 6B). This difference comes from both experimental error and differences between types of detection. The absorbance intensity measured in spectroscopic competition experiments reports LMCT mostly due to formation of Cd–S bonds. It should be remembered that not only tetrathiolate complexes are possibly formed and that even within the Cd(Cys)_4_ cores the intensities of LMCT may differ from each other because of the presence or absence of bridging bonds. Moreover, both types of experiments were performed at different reactant concentrations due to method requirements and this fact may also contribute to shifts in the constants. Nonetheless, stability data obtained from two significantly different methods are convergent and confirm a very wide range of Cd(II) affinity for GSH and PC peptides. They also clearly indicate trends in stabilities: increase from GSH to PC4 and a plateau above PC4, which was noted in pH-dependent spectroscopic titrations (Figure 4A,B). Interestingly, a comparison of apparent constants from the competition study with p*K*_a_′ values remains linear, indicating that in both types of experiments we observe the same phenomena (Figure 4C).^39^

### ITC Study

As discussed above, the ITC method demonstrates some drawbacks when it is applied for the investigations of metal-peptide/protein interactions.^38^ Its use for a complicated metal–ligand system with several species or application for determination of stability of highly stable complexes usually results in an underestimation of formation constants. Because of that fact, in this study, ITC experiments were performed solely qualitatively to examine the stoichiometry of the complexes formed and especially to compare the observable ITC enthalpies of Cd(II) complexation reactions (Δ*H*_ITC_) throughout the analyzed series of PCs.

**Figure 8:**
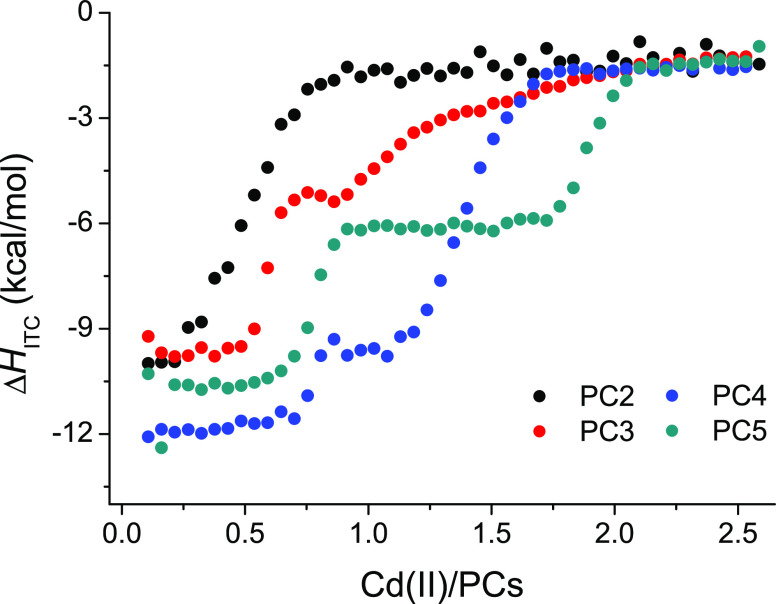
ITC results of Cd(II)-titrated phytochelatins (PC2–PC5) presented as function of the experimental enthalpy (Δ*H*_ITC_) and Cd(II)/PC molar ratio. All experiments were performed in HEPES buffer (*I* = 0.1 M from NaCl) at pH 7.4 with 3 mM TCEP used as a non-metal-binding reducing agent.^40,41^ The GSH or PC peptide (titrate) concentration was 250 or 50 μM, respectively, whereas the Cd(II) (titrant) concentration was 3 mM or 0.5 mM, respectively.

Previous efforts of applying ITC to study the thermodynamics of Cd(II) binding by phytochelatins, undertaken by the Esteban and the Ha-Duong groups,^29,33,34^ paint a confusing picture. First of all, thermodynamic parameters obtained during these studies are far from being uniform, which may be caused either by a frivolous incorporation of buffer deprotonation and complexation heats or by a complete lack of it. The other reason for the inhomogeneity of PC-related ITC data is the inherent and complex modularity of Cd(II)-binding processes that these peptides present. Every single injection of Cd(II) coincides with the generation of multiple Cd_*x*_(PCs)_*y*_ complexes in a dynamic equilibrium. Bearing in mind that phytochelatins interact with Cd(II) primarily via Cys thiolates and prefer tetrathiolate coordination spheres, one can safely assume that the thermodynamic effects of the aforementioned complex formation are probably very much alike. Moreover, phytochelatins constitute a group of short peptides without any tendencies to form highly ordered structures, even after complexation with Cd(II), which effectively negates any structural effect that would potentially diversify the ITC results of short and long PCs. All of the above suggests that the actual net enthalpy change should be comparable for the entire PC series. Figure 8 proves this assumption, as the overall Δ*H*_ITC_ change for the PC2–PC5 series varies only slightly.

Even though these ITC results show that the enthalpies of Cd(II) binding are comparable for phytochelatins from PC2 to PC5, there are still many discrepancies that have to be addressed. First of all, every single phytochelatin starting from PC2 shows a completely different isotherm of Cd(II) binding. Differences mainly pertain to the inflection of an isotherm and its slope, which correspond roughly with the stoichiometry and number of formed complexes and the affinity toward a given PC, respectively.

ITC data for Cd(II)-titrated phytochelatins illustrate stoichiometric preferences of Cd(II) complexes for different PCs. The inflection point of the isotherm sigmoidal curve and the molar ratio of Cd(II) to PCs gives an expected stoichiometry of the analyzed complex. The titration of PC2 is represented by an isotherm with a single inflection point at a Cd(II) to PC2 molar ratio of around 0.5 and suggests that under these experimental conditions the Cd(PC2)_2_ bis complex is preferentially formed. A similar bis-complexation tendency is observed for PC3; however, the one γ-Glu-Cys segment longer peptide gave two inflection points. The first point is situated at a molar ratio of around 0.6, though with a significantly larger slope in comparison with the PC2 isotherm, which correlates well with the increased affinity found in other experiments. It also confirms the presence of the Cd(PC3)_2_ complex that eluded detection during other experimental procedures. The other process, extending to a Cd(II) to PC3 molar ratio between 1 and 2, is significantly less resolved and much more prolonged. We suggest that the second isotherm is an effect of the formation of clustered species and the presence of multiple equilibria with intrapeptide-exchangeable Cd(II) ions. Between those two events a minor endothermic reaction is exposed that unveils itself as a small curve around a molar ratio of 0.7–0.8. It is possible that these heat alterations are generated by the formation of a Cd-PC3 monocomplex that harbors 3S donors and an additional nonsulfur donor, potentially responsible for the positive heat.^27^

PC4 begins the phytochelatin series with a fully equipped sulfur-donor binding site for Cd(II). This is emphasized on first glance by the higher negative value of the PC4 isotherm intercept that suggests additional enthalpic stabilization in comparison with shorter analogues. A PC4 titration shows two complex formation reactions—the first fitted to have an inflection point at ∼0.75 and the second at ∼1.4, which may be correlated with stoichiometries of 1 and 1.5 by assuming that the differences arose from overlapping processes, resulting in a shift of the inflection point. The first complex formation reaction in the isotherm plateaus at the PC2 and PC3 level and is characterized by a very high slope, indicating a substantial affinity increase in comparison with PC2 or PC3 (Figure 8). The second complexation process has a significantly lower slope, however, as is the case for PC2 (Figure 8). Similarities of the PC2 isotherm and the second isotherm of PC4 suggest that the second process recorded for the PC4 titration is connected with the formation of bis complexes throughout that particular range of Cd(II) to PC4 molar ratio. Moreover, the fitted stoichiometry and UV and CD spectroscopy results prove that these bis-complexes have the Cd_3_(PC4)_2_ structure.

The Cd(II) titration of PC5 is characterized by a biphasic isotherm, similarly to PC4 titration. The first reaction starts at the level of increased stability distinctive for longer PCs and plateaus at an enthalpic equilibrium that seems to be shared with PC3. The fitted stoichiometry is very similar to that of the PC4 result and equals a Cd(II) to PC ratio of approximately 0.75. Due to the fact that all longer phytochelatins exhibit stoichiometric values lower than those expected from spectroscopic and potentiometric results, we strongly suggest that this value is underestimated and is actually indicative of a 1:1 stoichiometry. Figure 8 shows that at the start of both isotherms an additional process takes place which bends the linear function before the inflection point. This tendency, also observed in the case of PC3, indicates the possible occurrence of an endothermic process that decreases the measured net enthalpy of Cd(II) binding. Interestingly, PC4 did not exhibit such behavior, which suggests that additional processes are somehow dependent on a surplus of free Cys thiols. The second complex formation reaction was fitted to a value of 1.86, and the shapes of both processes of the isotherm are very similar. These results demonstrate that at Cd(II) to PC ratios above 1.0, PC5 preferentially forms binuclear complexes with the Cd_*2*_PC5 structure. Cd(II) titrations of PC6 resulted in very complex thermograms with multiphase isotherms with no final equilibrium reached (data not shown). We propose that the cause of these intricate results pertains to the surplus of sulfur donors that increase the probability and number of clustered species formation.

## Discussion

### Cd(II)-GSH/PC Complex Speciation Profile

GSH and PC2–PC6 are highly dynamic systems in terms of the coordination chemistry. A multitechnique approach documents the stoichiometric relations of Cd–PC systems, thus providing evidence for the speciation-related studies never achievable with an application of even the most precise single-technique approach. Cd(II) forms with the investigated peptides various complexes, including bi-, mono-, and polynuclear species. The presence of myriad stoichiometries to some extent is derived from the high flexibility of PCs and lack of tendencies for secondary structure formation.

Cd(II) complexes with CdS_4_ binding modes, where terminal or bridged sulfur donors are present, predominate in solution, which is in line with the Cd(II) binding preferences. To warrant this requirement, the short PCs, i.e. PC2 and PC3, form bis complexes of a CdL_2_ fashion, with PC3 also forming clustered species such as Cd_2_L_3_ at higher Cd to PC3 molar ratios. Longer PCs fulfill the Cd(II) preferences and provide four sulfur donors, allowing metal ion sequestration with equimolar CdS_4_ species. Therefore, PC4–PC6 expose the high preference to CdL mono complexes at sub-equimolar metal concentration and form more complicated clustered species when the metal ion concentration exceeds the PC concentration (in the case of PC4 with a Cd_3_(PC4)_2_ stoichiometry and dimeric species for PC5 and PC6, respectively). Nevertheless, the presence of mixed-ligand species should not be ignored. Their formation allows for effective metal sequestration, yielding increased metal-buffering capacity that eliminates the possible detrimental consequences of Cd(II) interference with enzymatic systems involved in cell metabolism. For this reason GSH predominantly forms Cd(GSH)_2_ under the investigated conditions, while the Cd(GSH)_3_ and Cd(GSH)_4_ species are observed at much higher reactant concentrations.^73^ Following the same principle PC2 and PC3 form CdL complex species that, although without a fully thiolate coordination environment of Cd(II) ion, Cd{S_2_NO} and Cd{S_3_O}, respectively, predominate under equimolar conditions. A proportional increase of affinity toward the binuclear complex formation in the PC4–PC6 series related to the number of γ-Glu-Cys repeats is worth noting. Perhaps for that reason, PC5 and especially PC6 do not saturate at a Cd(II) to peptide molar ratio of 2.0, giving room for the formation of trinuclear species in solution. In such a case, a solely thiolate environment is not provided to all of the metallic nuclei of the complex. Furthermore, the increased flexibility of these complexes gives room for the formation of various polynuclear complexes or even polynuclear oligomers, postulated by others on the basis of chromatographic separation on natural PC sources.^18,21^ Even if they are not present to a great extent, their Cd(II) buffering capacity is greater due to the formation of metal sites with bridging sulfur donors.

### Thermodynamics of Cd(II)-PC Complex Formation

The results presented in the previous section show that in the peptide series from GSH to PC4 clear changes in the coordination modes of the ligands toward Cd(II) are observed, while close similarities are evident for PC4–PC6. One of the main questions that we wish to address and answer here is how the thermodynamic stability of Cd(II) complexes behaves in this series. Very little is known about the relationship between apparent formation/dissociation constants of Cd(II) complexes and the number of γ-Glu-Cys dipeptides in PCs. Recent articles in which authors used the ITC method to determine the stability constants of Cd(II) complexes with GSH and PCs show that these complexes barely differ from each other in terms of stability or the observable difference is smaller than expected.^29,34^ Moreover, the absolute constant value for the Cd-PC2 complex reported in the aforementioned articles was almost 4 orders of magnitude lower in comparison to potentiometric data obtained in the past and a current report.^27^ The major drawback of those articles, although they are filled with many useful observations, is the misapplication of the ITC method, which is known to underestimate stability constants of metal complexes due to either the method limitation (the maximum log *K* is 7–8 for direct titrations) or the wrong assumption that only one or two complexes are present, without a knowledge regarding their protonation states and the total number of reactions that should be taken into consideration.^29,34^ It has been explained step by step in our recent review what factors affect ITC experiments and their analysis or how ITC and other investigations should be performed to obtain the actual stability constants of metal complexes with peptides and proteins under the chosen conditions.^38,42^ Some other recommendations have also been underlined recently by Wilcox and colleagues.^81,82^ The fact that already reported ITC results in direct Cd(II) to thiol peptide titrations underestimated stability constants was one of our study’s major aims in which an examination of thermodynamics of the Cd-PC system was performed with care and application of convergent multitechnique analysis.

The first observation about major differences in affinities between Cd(II) complexes in the GSH and PC2–PC6 series was made here during pH-dependent spectroscopic titrations in which isotherms of complex formation were shifted by almost 2.5 orders of magnitude (Figure 4A,B). Such a major shift has been shown to be associated with several orders of magnitude difference in formation constants.^39,42^ Potentiometric data obtained in this report (Table 2) show that multiple complexes are formed, and their direct comparison without consideration of ligand protonation is impossible. In this situation, one can only roughly compare constants for the same complexes: e.g., all CdL or all CdHL formed per ligand (Figure 6A). Although those values are pH independent and are not valid for a particular pH, it is visible that the difference in stability between the least stable Cd(II) complex of GSH and the most stable complex is 12 and 14.5 orders of magnitude for CdL and CdHL complexes, respectively. Interestingly, this plot shows an almost linear increase in stability from GSH to PC4 and comparable constants for PC4 and PC5.

An evaluation of speciation profile and assigned stability constants over a wide pH range allowed us to translate them into more exoteric and widely appreciated competivity indexes that allow for a direct comparison between affinities assigned for different systems under different experimental conditions. The CI^7.4^ values determined show that GSH, PC2, and PC3 form Cd(II) complexes with micromolar, sub-nanomolar, and low-picomolar affinities, respectively. PC4–PC6 demonstrate similar femtomolar affinities toward Cd(II). The affinity difference between the weakest (GSH) and the strongest Cd(II) complexes (PC4, PC5) is more than 7 orders of magnitude in the formation constant, which transforms to a vast ΔΔ*G*° value of less than −10 kcal/mol, and this corresponds to an ∼−3.4 kcal/mol of stabilization Gibbs free energy effect per γ-Glu-Cys segment in the GSH-PC4 series.^42^

PC Cd(II) complexes with their wide range of stabilities can be compared with a set of various compounds, peptides, and proteins that bind Cd(II) by thiolate donors (Table 4).^42,55−58,83−89^ The weakest Cd–PC2 system’s stability is similar, for example, to that of a Cd(II) complex with a classical zinc finger domain, which offers two Cys residues in addition to two His residues.^84,89^ Cd(II) is a highly thiophilic metal ion, and an increase in sulfur donor number in the complex causes its stability to increase, which is very nicely demonstrated by a CP1 zinc finger series with two, three, and four Cys residues (Table 4). This does not explain why the stability of PC complexes changes so much within its series, while Cd(II) is bound by four Cys residues. It should be also noted that the stability increase is not caused by the metal-coupled folding process that contributes significantly to the enthalpy of the complexation. All PCs do not form stable secondary or tertiary structure folds upon Cd(II) complexation, as is for example observable in the case of CCCC CP1 ZF (femtomolar affinity) or an extremely stable Cd(II) complex with a zinc hook motif whose affinity is the highest observed to date (sub-zeptomolar affinity).^42,88^ A subtraction of metal-coupled processes results in Cd(II) binding to the tetrathiolate environment in the sub-nanomolar or picomolar range, which can be modulated by multiple effects depending on the complex.^57,58,90^ The reasons for the more than 6 orders of magnitude difference in stability with the PC series and almost 9 orders when one considers GSH cannot be explained without a deeper analysis of the complexation thermodynamics.

**Table 4:** Apparent Formation Constants and Competitivity Indexes of Cd(II) Complexes of Selected Thiol-Containing Ligands, Peptides, and Proteins^a^

ligand	log *K*^7.4^	CI^7.4^	ref
Hk130	nd	19.4	(42)
Hk14	nd	17.9	(39)
MT2 (α-cluster)	15.8	15.8	(57)
Ac-YCSSCY	nd	14.8	(83)
MT2 (β-cluster)	14.4	14.4	(57)
CP1 zf (CCCC)	13.4	13.4	(84)
XPA zf	12.8	12.8	(85)
DTBA	nd	12.8	(56)
Ac-CC-NH_2_	nd	12.6	(86)
CadC	12.6	12.6	(87)
Ac-EEGCCHGHHE-NH_2_	nd	12.5	(86)
CmtR	12.2	12.2	(88)
CP1 zf (CCCH)	11.2	11.2	(84)
DTT	nd	10.4	(55)
CP1 zf (CCHH)	8.7	8.7	(84)
TT-2D zf	8.5	8.5	(89)

a log *K*^7^^.^^4^, CI^7^^.^^4^, and nd stand for competitivity index, formation constant, and not calculated, respectively. log *K*^7^^.^^4^ values are not provided if stoichiometries of the formed complexes are other than ML or they were not determined in the original report. Abbreviations: DTBA, dithiobutanoic acid; DTT, dl-dithiotreitol. CI is the apparent dissociation constant of a CdL complex (Cd(II) complex of theoretical molecule Z), such as [CdZ] = ∑_*ijk*_[Cd_*i*_H_*j*_L_*k*_] at the given overall component concentrations. The concentrations of Z were set at 1 mM and those of Cd(II) at 0.25 mM.

The ITC results demonstrate that the molecular reasons for increased stability in the phytochelatin series are not due to enthalpy-related factors as suggested before. In that case, following the Gibbs equation, the Δ*G*° increase has to be correlated with a favorable entropic contribution of the system (eq 3). However, due to the inherent complexity of the Cd-PC system, i.e. dynamic equilibria with multiple clustered species formation, the ITC data were used solely qualitatively, with no intent to provide absolute values of Δ*H*°. Figure 8 shows that the observable heats of the entire PC series are comparable. Considering that the enthalpic contribution in the PC series is constant, yet the stability constants increase with the PC length, the free Gibbs energy decrease has to be connected with a positive change in the entropic factor (eq 3):
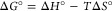
3There are two phenomena that shape the entropic landscape in the PC series and act in the opposite direction: (i) the chelate effect and (ii) conformational restriction. The chelate effect provides an entropic contribution for the longer PC as a direct outcome of stoichiometric and structural alterations and consequently from the various numbers of the substrates and products. However, the longer the PC is, the more restricted Cd(II) complex it forms. Thus, this conformational restriction provides an unfavorable entropic change to the system. Nonetheless, the overall energetic outcome suggests the major influence to be dictated by the chelate effect, which overshadows smaller energies of the latter process. Furthermore, the substantial boost in affinity established for PC4 and longer PC homologues may result from the peptides’ inherent capacity for the initial formation of tetrathiolate Cd(II) species, in contrast to the shorter PCs. We propose that these complexes are additionally stabilized in PC5 and PC6 by the entropic factor that originates from the higher accessibility of binding thiolates and the resulting structural flexibility of the cluster complex.

### Biological Significance

**Figure 9:**
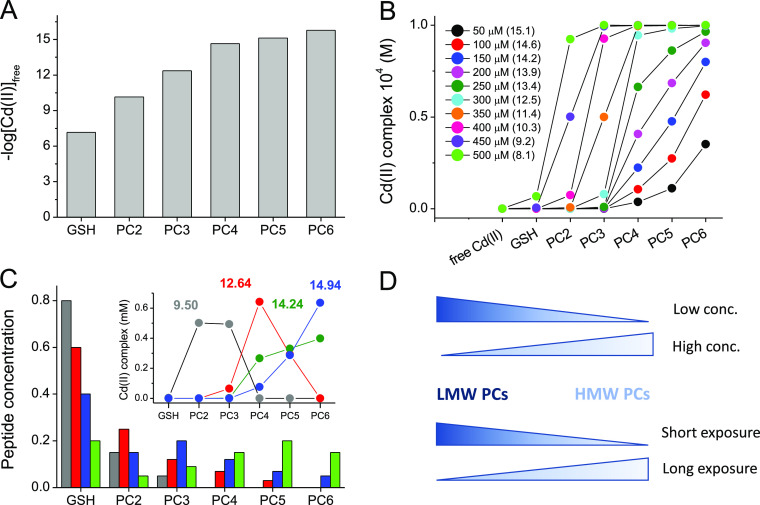
Speciation of Cd-L complexes and free Cd(II) concentrations in the series of GSHPC6. (A) Free Cd(II) concentrations a result of the complexation of 0.1 mM Cd(II) with 1 mM ligand. (B) Concentrations of Cd-L complexes in the system 0.5 mM GSH, 0.1 mM PC2–PC6, and increasing Cd(II) concentration from 50 to 500 μM (inset). Inset values in parentheses give free Cd(II) concentrations (−log[Cd(II)]_free_). (C) Various GSH and PC2–PC6 peptide concentration modeling dynamics of the γ-Glu-Cys peptide system during PC induction and related CdL complex concentrations in the presence of 0.1 M Cd(II). Case 1 (gray): GSH, PC2, PC3 concentrations are 0.8, 0.15, and 0.05 mM, respectively. Case 2 (red): GSH and PC2–PC5 concentrations are 0.6, 0.25, 0.12, 0.07, and 0.03 mM, respectively. Case 3 (blue): GSH and PC2–PC6 concentrations are 0.4, 0.15, 0.2, 0.12, 0.07, and 0.05 mM, respectively. Case 4 (green): GSH and PC2–PC6 concentrations are 0.2, 0.05, 0.09, 0.15, 0.2, and 0.15 mM, respectively. The inset demonstrates the distribution of CdL species in four investigated cases. Values in colors are log[Cd(II)]_free_ concentrations. (D) Scheme demonstrating speciation tendency for Cd(II) complexes of LMW and HMW PCs at low and high Cd(II) concentrations and under short and long exposures of Cd(II). LMW and HMW stand for low- and high-molecular weight peptides, respectively.

The results presented here show that the PC system demonstrates very interesting properties of Cd(II) buffering and detoxification that have not been presented and described in such detail to date. During heavy-metal exposure, plants and other PC-producing organisms start to change their sulfur metabolism in such a way that, from GSH, higher PCs are produced by conjugation of γ-Glu-Cys segments each by the other. At the same time GSH is produced, but the efficiency of the synthesis may be limited. Although the mechanism of PC biosynthesis is known, it is not clear how the production of PCs corresponds to their metal binding affinity and free Cd(II) concentration (cellularly available) that is required to keep this toxic metal unavailable to avoid any interference with biogenic metal ions such as Zn(II) or Cu(I). Those metal ions’ substitution could affect many cellular pathways and function of metalloproteins that rely on the biogenic metal ions. Cd(II) induction causes various PC production profiles in which the peptide ratio depends on the dose of the metal and the time of the exposure. Assuming that the exposure time increases Cd(II) concentrations in the cells, it becomes clear that Cd(II) must be detoxicated rapidly. Our results show that GSH does not have the capacity to bind Cd(II) tightly enough to avoid toxic consequences in the cell (micromolar affinity). The increased concentration of PC2 upon Cd(II) exposure significantly changes the buffering capacity of the γ-Glu-Cys system. The sub-nanomolar affinity and relatively fast induction of PC2 serve together as the first defense shield against Cd(II). An excess of PC2 over Cd(II) guarantees the formation of CdL_2_ complexes with the Cd(Cys)_4_ core and keeps free Cd(II) at relatively low free concentrations. Figure 9A shows a simulation based on the Cd(II) affinities of the GSH and PC system buffering, indicating ranges in free Cd(II) in the presence of particular peptides alone. This tendency is highly similar to that observed for the comparison of stability constants obtained in this study, showing that the GSH and PC2–PC6 series buffering properties change from the micro- to femtomolar range presented as a free Cd(II) change. The increase of Cd(II) in a cell results in the appearance of PC3, which more tightly binds metal ions, lowering their cellular availability. Longer PCs are synthesized during more extensive induction and time of exposure and are, according to many analytical investigations, present together with shorter PCs. Figure 9B presents the Cd(II) speciation of a peptide mixture, where GSH and PC concentrations are 0.5 and 0.1 mM, respectively, while the Cd(II) concentration varies from 0.05 to 0.5 mM. It clearly shows how fractions of particular complexes change in the total Cd(II) increase. A low metal concentration results in the formation of PC6, PC5, and PC4 Cd(II) complexes due to their highest affinity. When the concentration of total Cd(II) increases and longer PCs become saturated, the system starts to use PC3 and then PC2 and finally GSH to bind Cd(II) at much higher free metal concentrations. In the cell fractions of particular peptides changes in time and metal exposure indicate the dynamics of the system. Figure 9C demonstrates four various scenarios where GSH, PC2, PC3, and PC5 dominate over other GSH and PC2–PC6 peptides (relative peptide molar fractions), while the inset shows what in fact occurs with Cd(II) under these conditions. Interestingly, in none of the cases is GSH a significant Cd(II) ligand, even when it dominates over PCs, indicating its different role in Cd(II) detoxification. Depending on the considered case, particular PCs play an important role in Cd(II) binding—short PCs when they dominate and longer PCs if they are present at a more significant level. Importantly, in all cases free Cd(II) concentrations vary from the picomolar to low-femtomolar range, illustrating that this metal ion is very well chelated under dynamic conditions. Figure 9D summarizes the coordination dynamics of the system discussed here, indicating the importance of gradually changed affinities toward Cd(II). Depending on the metal concentration and time of cell exposure the roles of particular PCs are different but critical for the whole buffering system.

It is paramount to address that Cd(II) is not the only inducer of phytochelatin synthesis. PC synthase may also be triggered by Pb(II), Zn(II), Sb(III), Ag(I), Ni(II), Hg(II), Cu(II), Sn(II), Au(I), Bi(III), AsO_4_^3–^, and SeO_3_^2–^, as well as platinum-group elements, such as Pt(II), Pd(II) and Rh(II), that present a significant environmental impact as pollutants.^14−16^ Though our work was dedicated to evaluating the most efficient inducer of PC synthesis that is Cd(II), and therefore cannot be directly translated to other elements, it provides a biophysical background for a further investigation and potential application of model plants in the bioremediation of the listed pollutants. Our studies show that PCs, although they have been studied for almost 40 years now, are vastly complex and further studies are needed to fully understand all the peculiarities of speciation, structure, and stability of this *terra incognita* of PC metal ion complexes.

## Conclusions

To summarize, data presented here show that GSH and PC2–PC6 are highly dynamic systems in terms of the coordination chemistry. Cd(II) forms with the investigated peptides bi-, mono-, and polynuclear complexes in tetrathiolate cores, where terminal or bridged sulfur donors are present. Only GSH and PC2 with excess metal form other binding modes. The presence of so many stoichiometries derives from the various Cys residues present in the particular PCs, their high flexibility, and lack of tendencies for secondary structure formation. A thermodynamic analysis showed the Cd(II) affinity to PCs with a large range of affinities from micro- to femtomolar, which has not been demonstrated to date. The data show that this large complex stability increase occurs almost exclusively from GSH to PC4, and above that (PC5 and PC6) it is almost constant, with a minor increase. A calorimetric investigation demonstrated that the observed stability elevation is not driven enthalpically but entropically, mostly due to the formation of various stoichiometries of complexes from the PC2–PC4 series and related macrochelate effects. Our results also show an important effect of multinuclear sites, especially in higher PC forms. Even if they are not present to a great extent, their Cd(II) buffering capacity is greater due to the formation of metal sites with bridging sulfur donors. This results in a more efficient use of higher PCs while keeping Cd(II) buffering and its free concentration retained. Data and the performed simulation show that despite Cd(II) influx the cell keeps its free concentration very low by two different mechanisms: one relies on increased metal to peptide affinity with the GSH and PC2–PC4 series and the other onthe more efficient complexation of PCs above PC4. Entropy actually drives both processes, but to different degrees. Keeping Cd(II) at a very low available level is achieved by high PC relative changes (biosynthesis) and coordination dynamics. This allows cells to handle various quantities of toxic metal ions and avoid interference with biogenic metal ions such as Zn(II).

## Supporting Information Available

Protocol for peptide synthesis with eexperimental and theoretical monoisotopic mass values; Differential absorption spectra of GSH and PCs titrations in UV range, schematic representation of selected Cd(II) complexes formed by PC2–PC4, ESI-MS spectra of PC2–PC6 complexes, distribution of fractionally ionized forms of GSH and PCs determined potentiometrically, binuclear complex speciation for Cd(II) complexes of PC3–PC5 at a Cd(II) to peptide ratio of 2.0, protonation and Cd(II) stability constants of NDAP and TPP, molar species distribution of Cd(II) complexes with NDAP, and relation of total and free Cd(II) concentrations for chelating agents used in this study at pH 7.4 (PDF)
