# Supplementary material for: Phytochelatins as a Dynamic System for Cd(II) Buffering from the Micro- to Femtomolar Range
Source: Inorg Chem. 2021 Mar 19;60(7):4657–75. doi: 10.1021/acs.inorgchem.0c03639 (PMC8041291; doi:10.1021/acs.inorgchem.0c03639)
Supplement: Supplementary file 1 — ic0c03639_si_001.pdf [file ic0c03639_si_001.pdf]

Supplementary information

Phytochelatins as a dynamic system for Cd(II) buffering from micro- to femtomolar range

Joanna Wątły,[†] Marek Łuczkowski,[†] Michał Padjasek and Artur Krężel*

Contribution from

Department of Chemical Biology, Faculty of Biotechnology, University of Wrocław, Joliot-Curie 14a, 50-383 Wrocław, Poland

† These authors contributed equally to this work

Correspondence to Artur Krężel

E-mail: artur.krezel@uwr.edu.pl

Table of content

Peptide synthesis……………………………………………………………………………...S3

Table S1. Experimental and theoretical monoisotopic mass values of synthesized peptides and their sequences. ……………………………………………………………………..…...….. S4

Figure S1. Differential absorption spectra of GSH (100 μM), PCs (20 μM), and PC3-PC6 (10 μM) titrations in UV range.……………………………………………………………….….S5

Figure S2. Schematic representation of selected Cd(II) complexes formed by PC2-PC4 with the indication of complex stoichiometry. …………………………………………………………...S6

Figure S3. ESI-MS spectra of 50 μM PC2 peptide.………………………………………………..S7

Figure S4. ESI-MS spectra of 50 μM PC3 peptide. …………………………………………….…S8

Figure S5. ESI-MS spectra of 50 μM PC4 peptide. …………………………………………….…S9

Figure S6. ESI-MS spectra of 50 μM PC5 peptide. …………………………………….………..S10

Figure S7. ESI-MS spectra of 25 μM PC6 peptide. …………………………………….……….S11

Figure S8. Distribution of fractionally ionized forms of GSH, PC2 and PC3 determined potentiometrically. …………………………………………………………………………….S12

Figure S9. Distribution of fractionally ionized forms of PC4, PC5 and PC6 determined potentiometrically. …………………………………………………………………………….S13

Figure S10. Species distribution profiles for Cd(II) complexes of GSH. …………………...S14

Figure S11. Binuclear complexes speciation for Cd(II) complexes of PC3, PC4 and PC5 at Cd(II)-to-peptide ratio of 2.0 (1 mM Cd(II): 500 μM PCs). ……………………………..….S15

Table S2. Protonation and Cd(II) stability constants of NDAP and TPP. …………………..….S16

Figure S12. Molar species distribution of Cd(II) complexes with NDAP. …………………S17

Figure S13. Relation of total and free Cd(II) concentrations for chelating agents used in this study at pH 7.4. ………………………………………………………………………………..S18

Peptide synthesis. Phytochelatin synthesis was performed on a solid state support using the Fmoc strategy. For that purpose Wang resin with preloaded Gly was used to introduce the first C-terminal amino acid residues. Fmoc-Cys(Trt)-OH was used in the whole protocol to elongate the peptide chain. Our previous observation showed that this amino acid derivative works very well for glutathione derivative synthesis.[1] Glutamic acid with the gamma peptide was introduced using Glu(OH)-OtBu (Merck), which exclusively forms a peptide bond with the gamma carboxylate group from the C-end instead of standard α-carboxylate. Synthesis of particular (γ-Glu-Cys)$_n$-Gly peptides (PCs) was performed so that a greater amount of resin was divided during synthesis starting from $n = 2$ to obtain equimolar amounts of PC peptide. If necessary, a particular peptide was synthesized solely. GSH (γ-Glu-Cys-Gly) was obtained from a commercial vendor as a high-quality peptide. Scheme 1 presents sequences of all investigated peptides with assigned names used in this work.

Table S1. Experimental and theoretical monoisotopic mass values of synthesized peptides and their sequences.

Peptide name	Peptide sequence	MW_{cal}	MW_{exp}
PC2	γECγECG	539.1	539.3
PC3	γECγECγECG	771.2	771.3
PC4	γECγECγECγECG	1003.2	1003.3
PC5	γECγECγECγECγECG	1235.3	1235.6
PC6	γECγECγECγECγECγECG	1467.3	1467.6

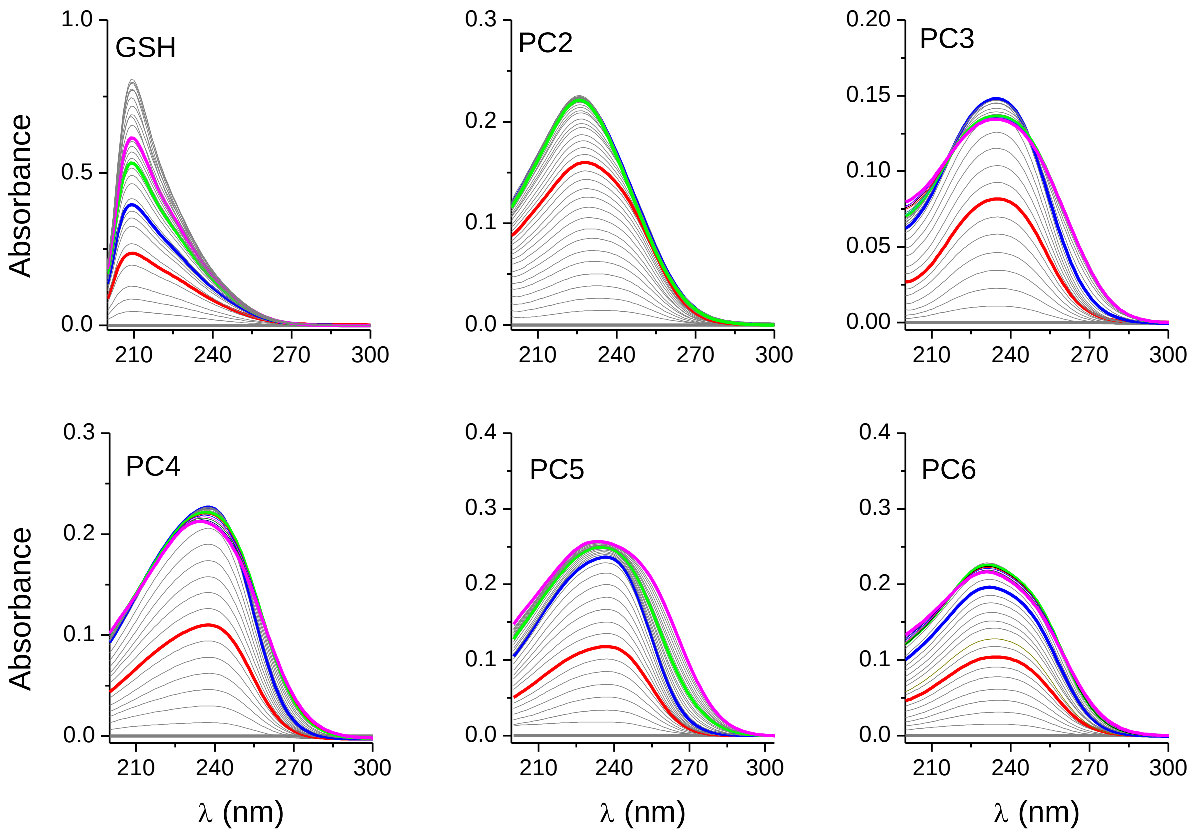

Figure S1. Differential absorption spectra of GSH (100 μM), PCs (20 μM), and PC3-PC6 (10 μM) titrations in UV range derived from Figure 1. Grey, red, blue, green, and magenta color of the spectra correspond to Cd(II)-to-peptide ratio of 0, 0.5, 1, 1.5 and 2, respectively. Spectra were recorded in 20 mM Tris-HCl buffer at pH 7.4 (I = 0.*1* M from $NaClO_4$).

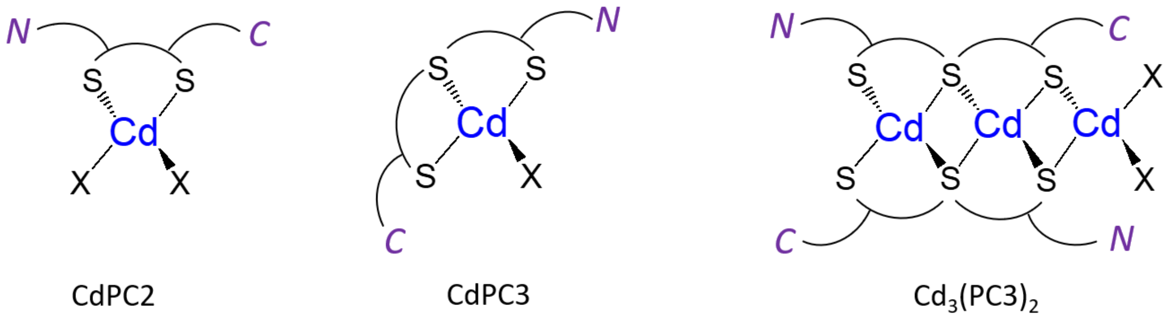

Figure S2. Schematic representation of selected Cd(II) complexes formed by PC2-PC3 with the indication of complex stoichiometry. *N* and *C* denote N-terminus (γ-Glu) and C-terminus (Gly residues) of each phytochelatin. Note that C and N-termini may be protonated or deprotonated. X represents either donors from N- or C-terminus or water molecule that fill coordination sphere of Cd(II).

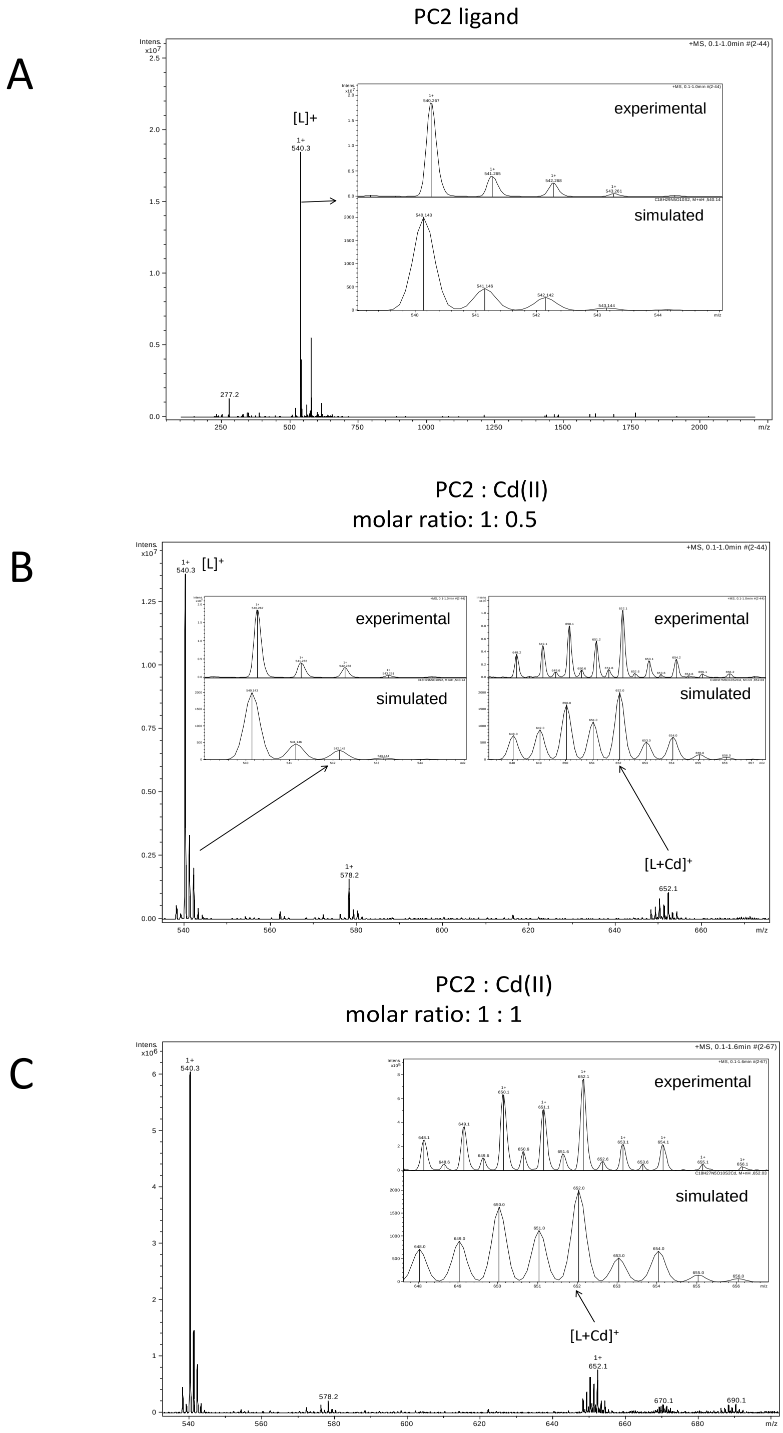

Figure S3. ESI-MS spectra of 50 μM PC2 peptide prepared in 10 mM NH_4HCO_3 for(A) free ligand (B) and (C) mixtures of Cd(II)-to-PC2 at molar ratios 0.5 and 1.0, respectively.

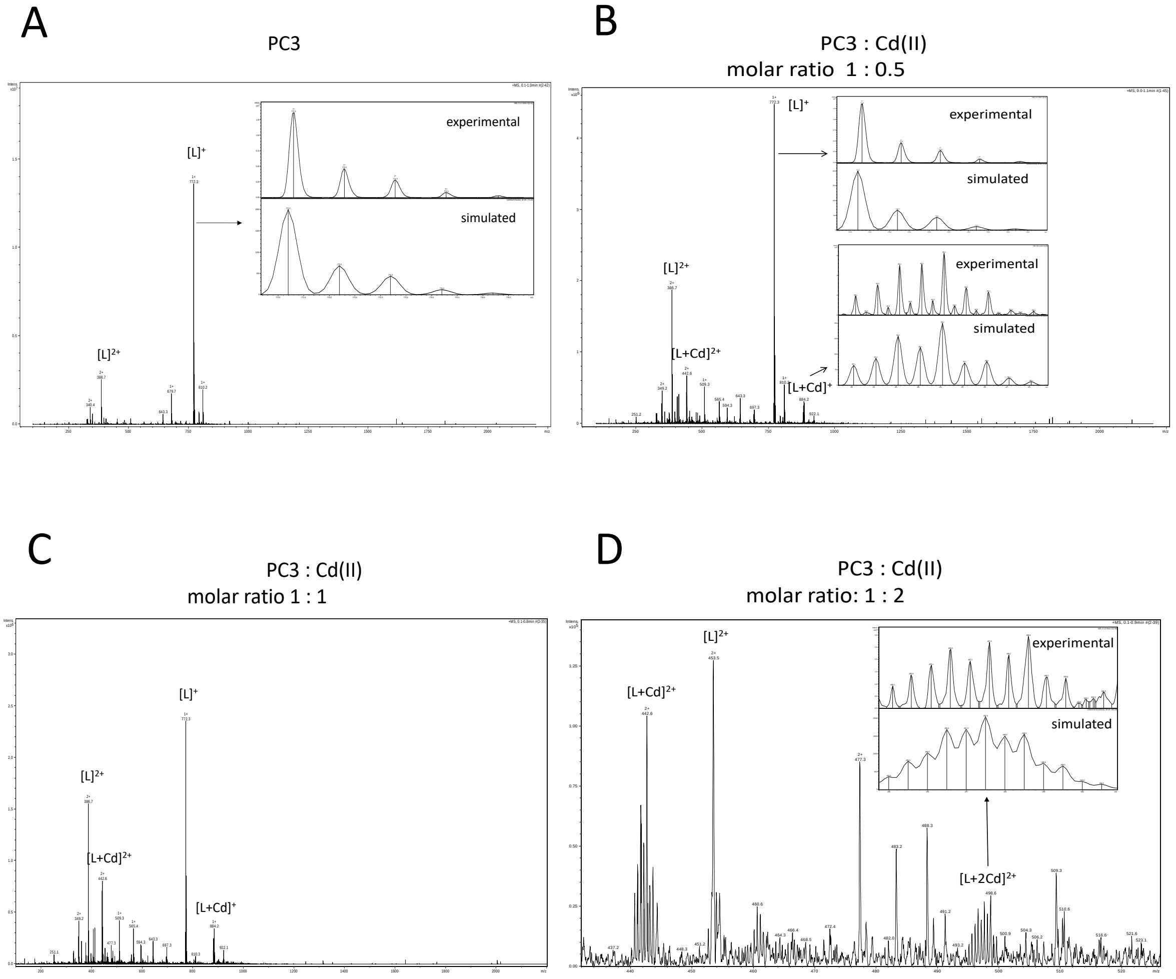

Figure S4. ESI-MS spectra of 50 μM PC3 peptide prepared in 10 mM NH_4HCO_3 for (A) free ligand (B) –(D) mixtures of Cd(II)-to-peptide complexes at molar ratios 0.5, 1.0, and 2.0, respectively.

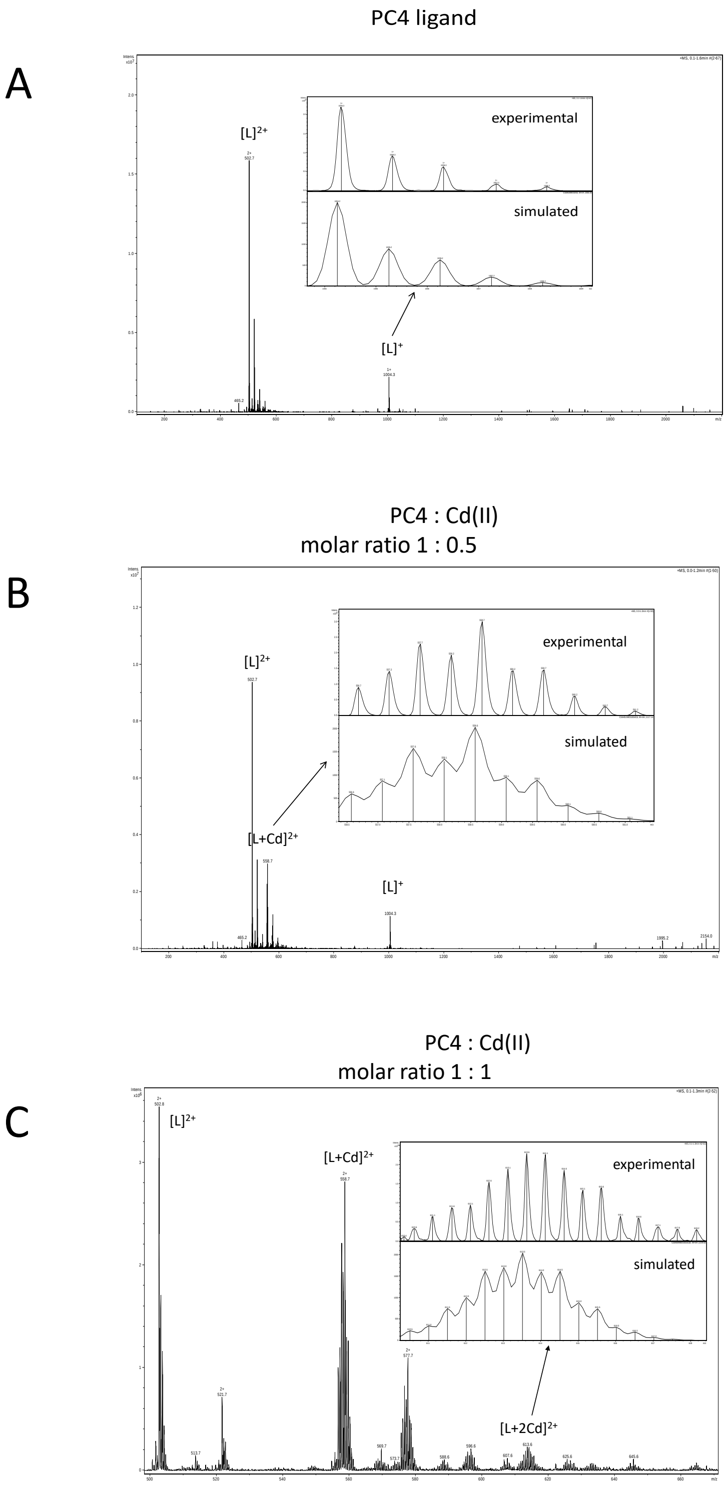

Figure S5. ESI-MS spectra of 50 μM PC4 peptide prepared in 10 mM NH_4HCO_3 for (A) free ligand (B) and (C) mixtures of Cd(II)-to-peptide at molar ratios 0.5 and 1:1, respectively.

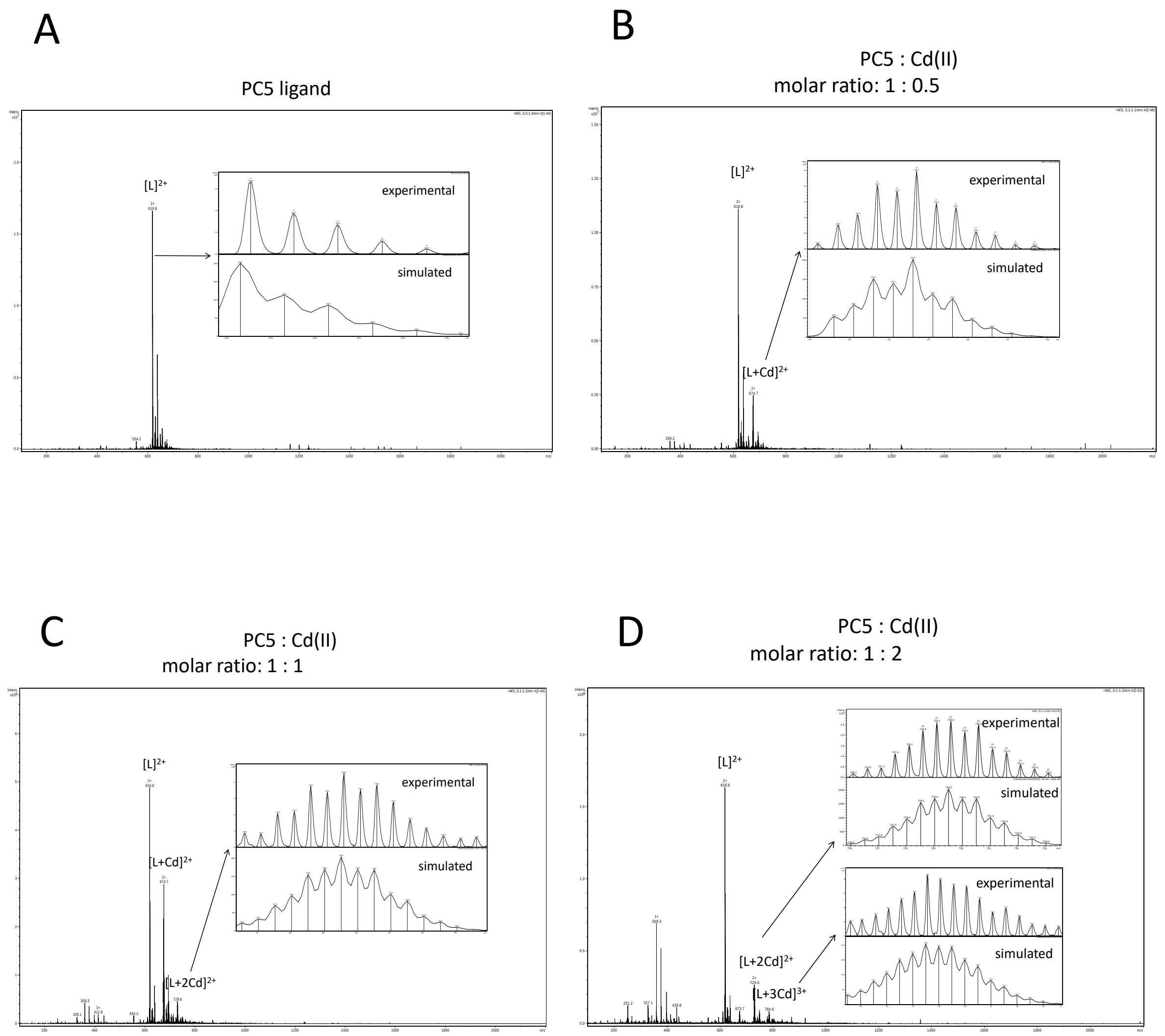

Figure S6. ESI-MS spectra of 50 μM PC5 peptide prepared in 10 mM NH_4HCO_3 for (A) free ligand (B) –(D) mixtures of Cd(II)-to-peptide at molar ratios 0.5, 1.0, and 2.0 respectively.

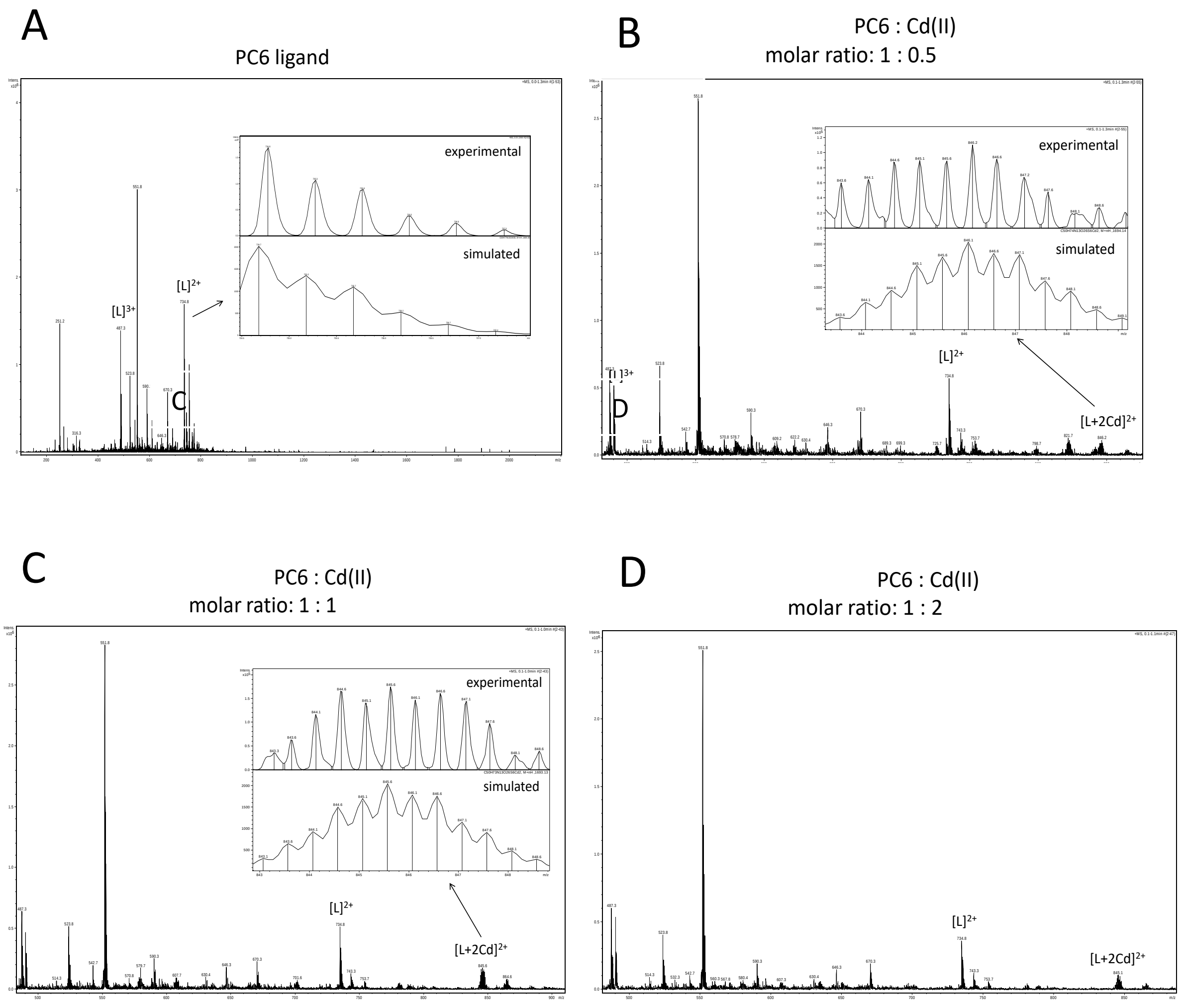

Figure S7. ESI-MS spectra of 25 μM PC6 peptide prepared in 10 mM NH_4HCO_3 for (A) free ligand (B) –(D) mixtures of Cd(II)-to-peptide at molar ratios 0.5, 1.0, and 2.0, respectively.

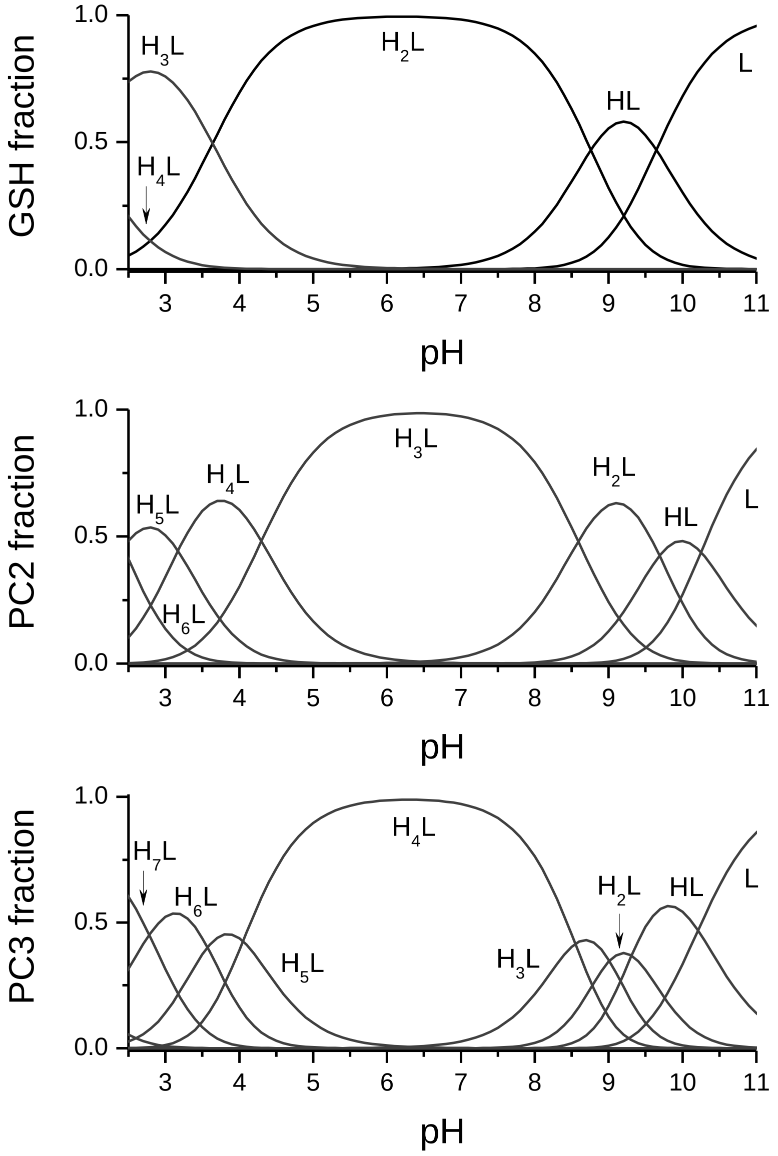

Figure S8. Distribution of fractionally ionized forms of GSH, PC2 and PC3 determined potentiometrically (I = 0.1 M from KNO_3). Original data presented in Table 1.

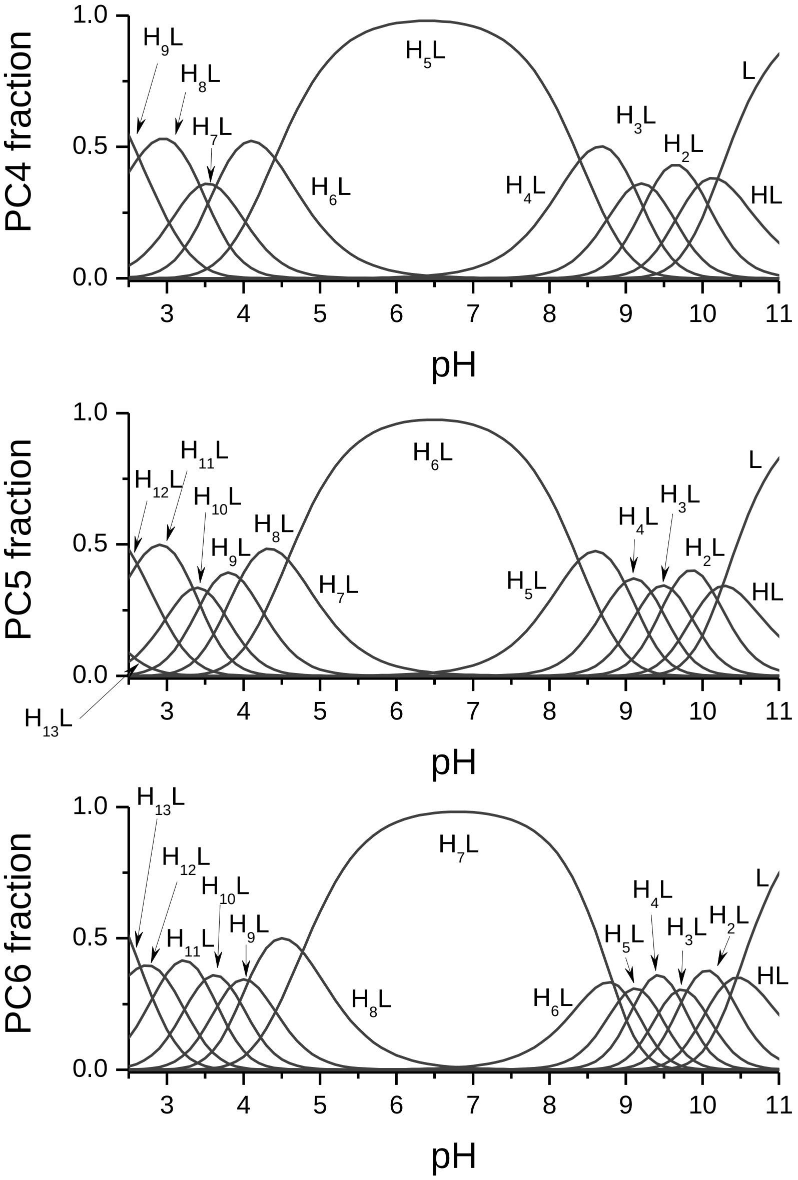

Table S9. Distribution of fractionally ionized forms of PC4, PC5 and PC6 determined potentiometrically (I = 0.1 M from KNO_3). Original data presented in Table 1.

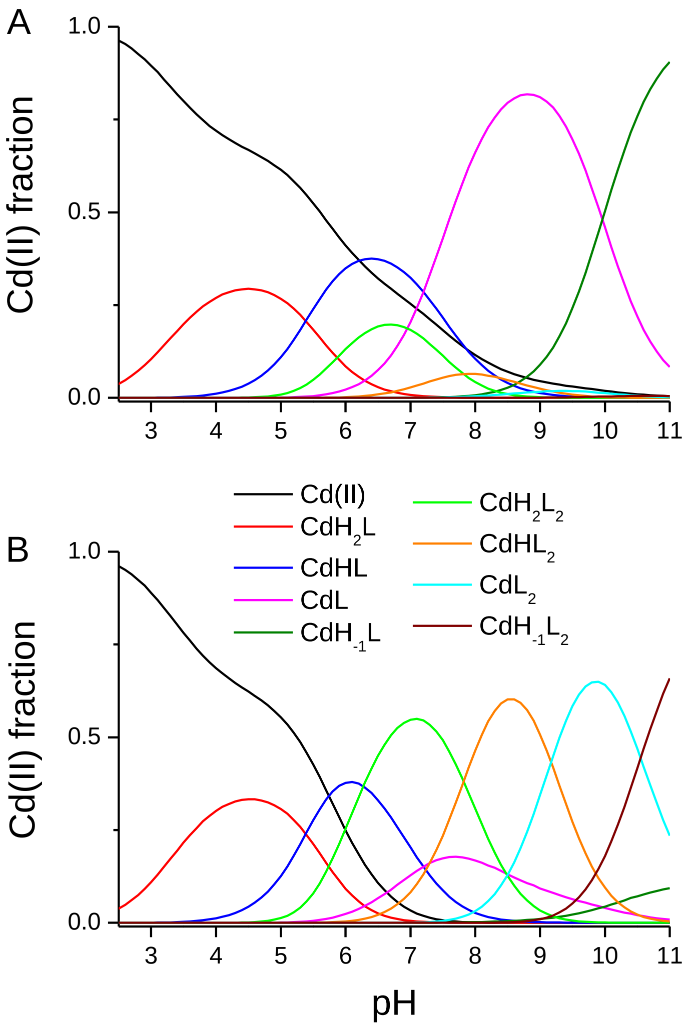

Figure S10. Species distribution profiles for Cd(II) complexes of GSH at 1:1 (A) and 1:2 (B) Cd(II)-to-peptide molar ratio 1.0. Concentration of GSH in both simulations was 500 μM. Cd(II) : 500 μM PCs) based on potentiometric results (25°C, *I* = 0.1 M from KNO_3). Original data presented in Table 1 and Table 2.

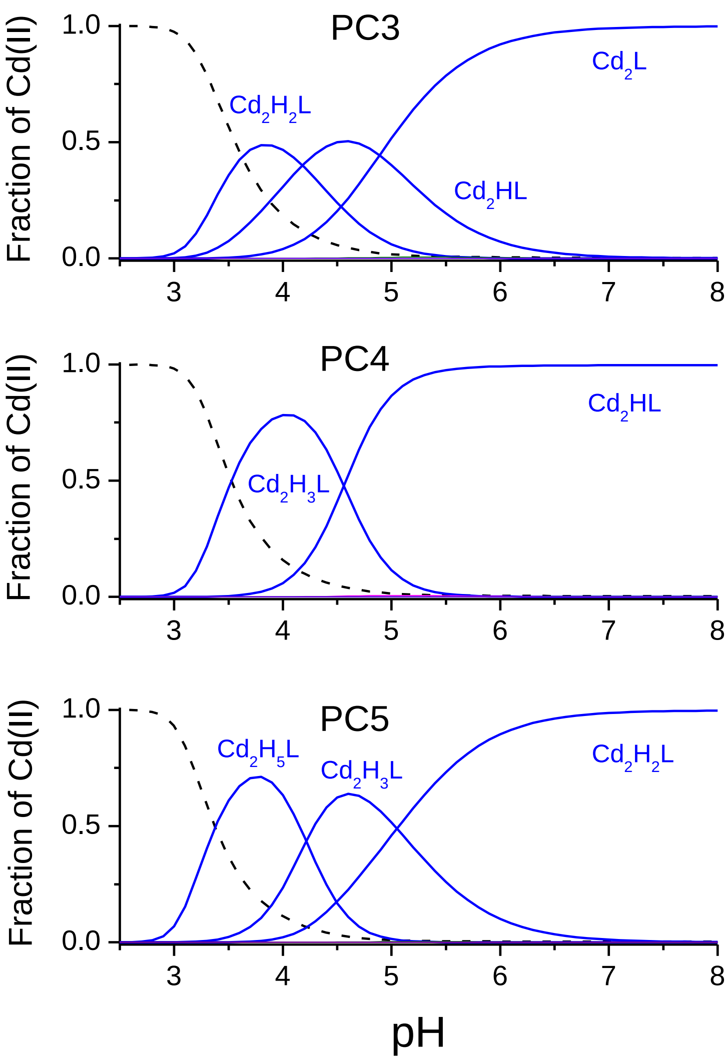

Figure S11. Binuclear complex speciation for Cd(II) complexes of PC3, PC4 and PC5 at Cd(II)-to-peptide ratio of 2.0 (1 mM Cd(II): 500 µM PCs). Diagrams are plotted to pH 8 since their deprotonation processes are unknown due to coexistence with mononuclear complexes which dominate under conditions used in potentiometric titration. Dashed line presents free Cd(II). Color and style of the figure correspond to Figure 5 in the main text. Original data are presented in Table 1 and Table 2.

Table S2. Protonation and Cd(II) stability constants of NDAP and TPP at 25°C (I = 0.1 M from KNO_3). Constants are presented as cumulative logβ_{ijk}ones.[a]Values in italics correspond to pK_a values of the ligands and were derived from cumulative constants.[b]

species	logβ_{ijk}	
	NDAP	TPP
HL	9.61 ±0.02*9.61*	7.93±01*7.93*
H_2L	13.29 ±0.03*3.68*	13.35±0.02*5.42*
H_3L	15.08±0.04*1.78*	-
CdHL	11.99 ±0.06*1.30*	12.41±0.01*2.67*
CdL	8.29 ±0.01*3.70*	6.99±0.01*5.42*
$CdH_{-1}L$	-2.20 ±0.02*10.49*	-3.81±0.02*10.80*

[a] $\beta(M_iH_jL_k)=[M_iH_jL_k]/([M]^i[H]^j[L]^k)$, in which [L] is the concentration of the fully deprotonated ligand.

[b] $\log\beta(H_jL_k) - \log\beta(H_{j-1}L_k) = \mathrm{p}K_a$.

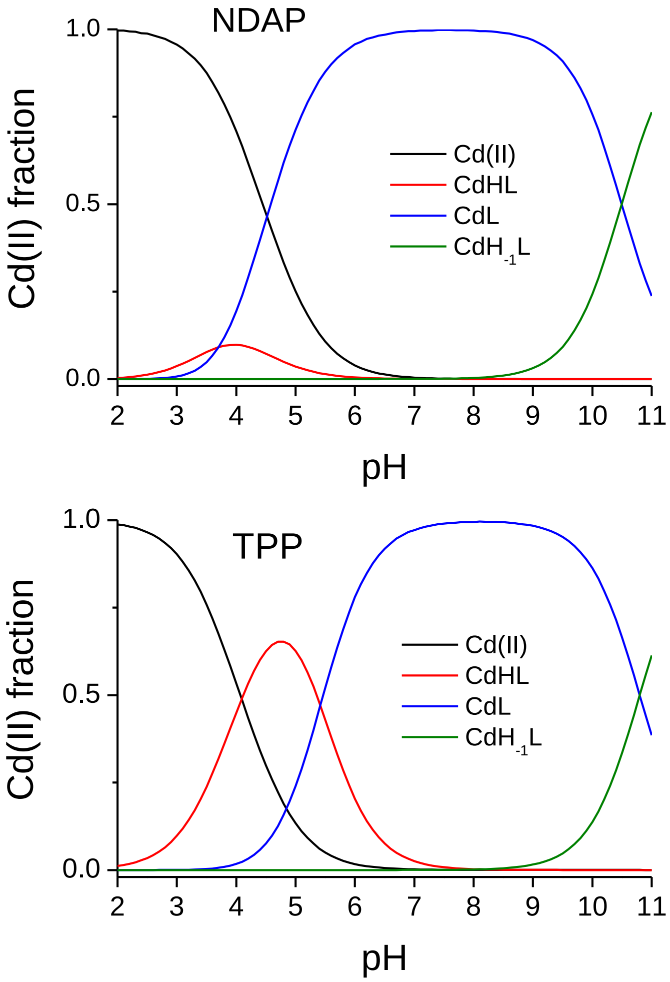

Figure S12. Molar species distribution of Cd(II) complexes with NDAP (top) and triphosphoric acid (TPP, bottom) calculated based on protonation and stability constants determined potentiometrically in this study. Ligand and metal ion concentrations were set as 1.0 and 0.5 mM, respectively.

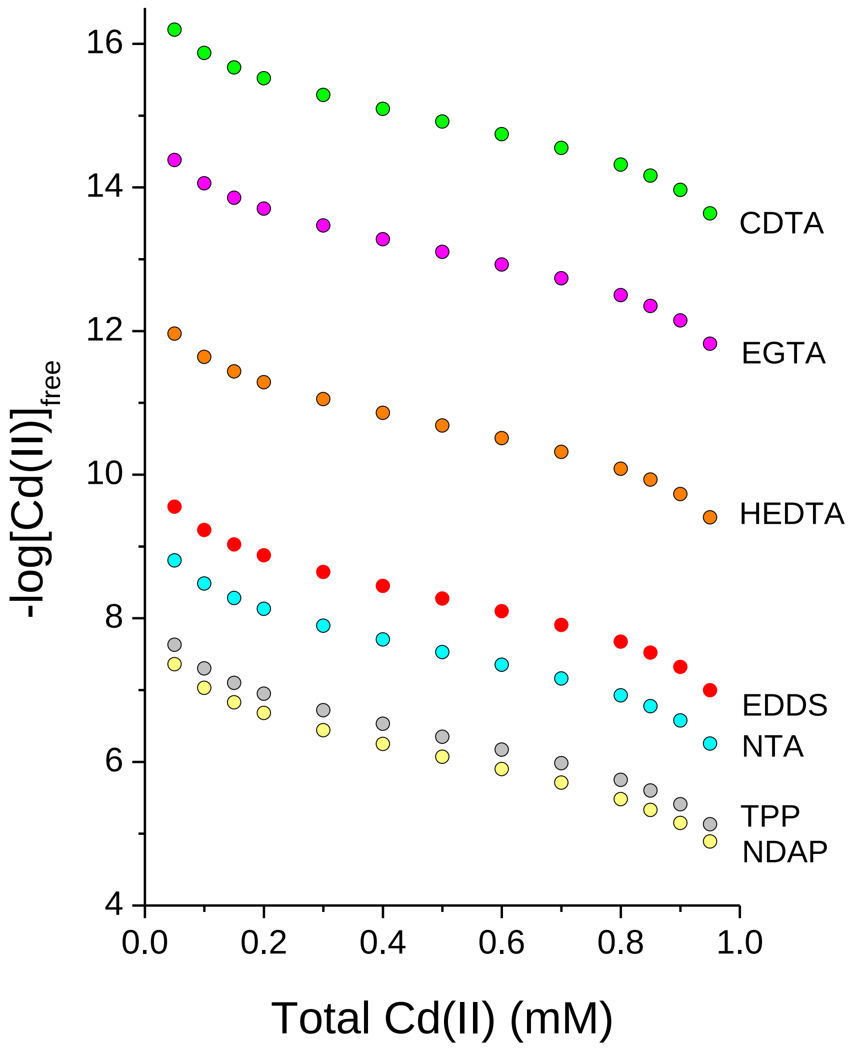

Figure S13. Relation of total and free Cd(II) concentrations for chelating agents used in this study at pH 7.4 (I = 0.1 M). The -log([Cd(II)]$_{free}$) values were calculated for 1 mM chelating component and 0.05 – 0.95 mM Cd(II).

REFERENCES:

1. Krężel, A.; Bal, W. Structure-function relationships in glutathione and its analogues. Org. Biomol. Chem.2003, 1, 3885−3890.
